# Isostructural behaviour in ammonium and potassium salt forms of sulfonated azo dyes

**DOI:** 10.1107/S2053229624001293

**Published:** 2024-02-15

**Authors:** Alan R. Kennedy, Jennifer B. A. Kirkhouse, Karen M. McCarney, Olivier Puissegur

**Affiliations:** a Department of Pure & Applied Chemistry, University of Strathclyde, Glasgow, G1 1XQ, United Kingdom; Universidade Federal de Minas Gerais, Brazil

**Keywords:** single-crystal X-ray diffraction, dye, salt selection, sulfonated azo, pseudo-alkali metal, alkali metal, crystal structure, isostructural

## Abstract

The structures of five ammonium salt forms of mono­sulfonated azo dyes, derivatives of 4-(2-phenyldiazen-1-yl)benzenesulfonate, with the general formula [NH_4_][O_3_S(C_6_H_4_)NN(C_6_H_3_)*RR*′]·*X*H_2_O [*R* = OH, NH_2_ or N(C_2_H_4_OH)_2_; *R*′ = H or OH] are presented. All form simple layered structures with alternating hydro­phobic (organic) and hydro­philic (cation, solvent and polar groups) layers. To assess for isostructural behaviour of the ammonium cation with *M* ^+^ ions, the packing of these structures is compared with literature examples. To aid this comparison, the corresponding structures of four potassium salt forms of the mono­sulfonated azo dyes are also presented herein. Of the five ammonium salts it is found that three have isostructural equivalents. In two cases this equivalent is a potassium salt form and in one case it is a rubidium salt form. The isostructurality of ion packing and of unit-cell symmetry and dimensions tolerates cases where the ammonium ions form somewhat different inter­action types with coformer species than do the potassium or rubidium ions. No sodium salt forms are found to be isostructural with any ammonium equivalent. However, similarities in the anion packing within a single hydro­phobic layer are found for a group that consists of the ammonium and rubidium salt forms of one azo anion species and the sodium and silver salt forms of a different azo species.

## Abstract 2

Of five ammonium salt forms of sulfonated azo dyes, three are found to be isostructural with either their K or Rb congeners.

## Introduction

The process of salt selection aims to choose the form of an active organic material that has the best properties for effectiveness and for commercialization. Salt selection is well studied in the area of pharmaceuticals (Stahl & Wermuth, 2008 ▸; Mahmood *et al.*, 2023 ▸; Bharate, 2021 ▸; Arlin *et al.*, 2011 ▸; Black *et al.*, 2007 ▸), but it is perhaps less well known that similar processes are used to select for material properties in other areas too. One example is the process of laking sulfonated azo colourants. A typical process here involves the substitution of an *M* ^+^ cation with an *M* ^2+^ cation, such as Ca or Ba, to switch from an aqueous-soluble dyestuff to an insoluble pigment (Christie & Mackay, 2008 ▸; Schmidt *et al.*, 2009 ▸; Kennedy *et al.*, 2012 ▸). As with pharmaceuticals, the material properties of pigments are dependant upon their crystal structures, and upon the inter­molecular inter­actions present within the crystal (Hao & Iqbal, 1997 ▸). However, in the field of sulfonated azo colourants relatively few pigment structures are known due to the insoluble nature of the materials and to the highly anisotropic habits of many species. This means that a high percentage of the crystal structures that are known are derived from less common methods than standard single-crystal X-ray diffraction (*e.g.* structure from powder diffraction, from electron diffraction or through use of synchrotron radiation – see Schmidt *et al.*, 2009 ▸; Gorelik *et al.*, 2009 ▸; Kennedy *et al.*, 2000 ▸; Grzesiak-Nowak *et al.*, 2019 ▸). One strategy for understanding the structure of sulfonated azo pigments has been to study systematically the structures of similarly functionalized, but easier to manipulate, dyes and then to cross-check any structure-to-property relationships identified against those pigment structures that are known (Kennedy *et al.*, 2004 ▸, 2009 ▸, 2012 ▸).

Ammonium salt forms of sulfonated azo colourants are sometimes used in preference to alkali-metal salts, either in the finished product or as an inter­mediate prior to laking (Christie & Mackay, 2008 ▸; Al Isawi *et al.*, 2021 ▸; Gonzalez & Miksovska, 2014 ▸). Despite this, only two structures of ammonium salts of sulfonated azo colourants appear to have been determined, namely, those of di­ammonium Orange G tetra­hydrate and of the nitrile-substituted [NH_4_][O_3_S(C_6_H_4_)NN(C_6_H_4_)NHCH_2_CH_2_CN]·H_2_O (Ojala *et al.*, 1994 ▸; Astbury *et al.*, 2013 ▸). The NH_4_^+^ ion is sometimes known as a pseudo-alkali metal due to it propensity to act in an isostructural manner with the heavier Group 1 metal ions. Its effective ionic radius has been estimated at 1.40 to 1.67 Å, depending on the coordination number (Sidey, 2016 ▸). Thus, as well as having the same charge as an alkali metal, it also has an ionic radius simi­lar to those of K^+^ and Rb^+^. An obvious difference is that bonds from an alkali-metal ion to, for example, an O-atom donor are typically described as ionic *M*—O inter­actions, whereas NH_4_^+^ will inter­act with O *via* N—H⋯O hydrogen bonds. As there are four H-atom donors per ammonium ion this may limit NH_4_^+^ to lower coordination numbers than those typically seen for K or Rb. It seems to be this feature that is responsible for ammonium forming isostructural pairs with Na compounds, as well as with K and Rb compounds, despite the smaller ionic radius of Na^+^ (*e.g.* Khan & Baur, 1972 ▸; Christov, 2003 ▸; Emerson *et al.*, 2014 ▸). Many examples of isostructurality between ammonium and Group 1 metal ions are for inorganic systems, but organic examples are also known. Of particular relevance to sulfonated azo species is the sweetener cyclamic acid. This is an *R*SO_3_^−^-containing organic species and its ammonium, Na, K and Rb salt forms are known to form an isostructural series (Leban *et al.*, 2007 ▸).

In order to investigate the structural relationships between ammonium salt forms of sulfonated azo dyes and their alkali-metal congeners, we herein present the crystal structures of ammonium salts of five azo anion species (Scheme 1). The structures of their Na-salt equivalents have already been reported (Kennedy *et al.*, 2001 ▸, 2020 ▸; Dodds *et al.*, 2017 ▸), as has the structure of one of their K equivalents (Kennedy *et al.*, 2004 ▸). In order to complete the comparison we report herein the crystal structures of the remaining four K-salt equivalents. 
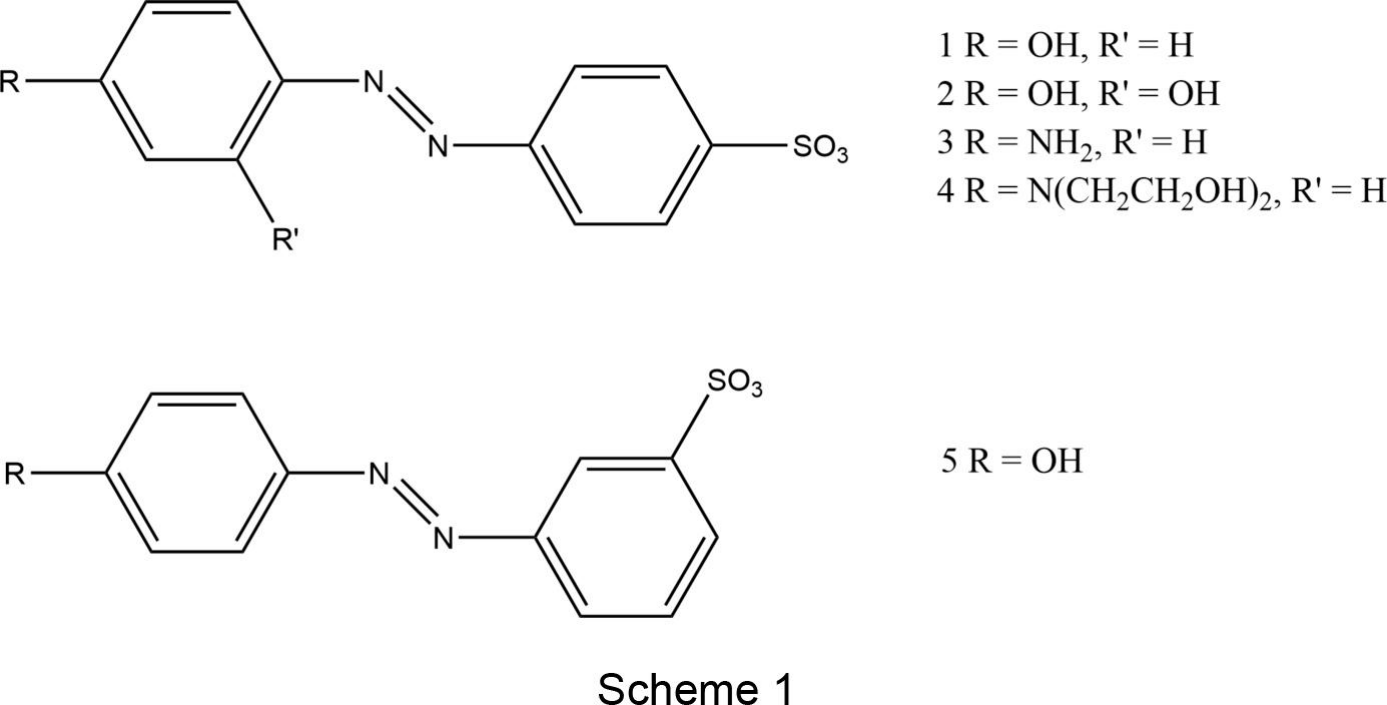


## Experimental

The Na salt of dye **3** was obtained from Fujifilm. The other dyes were synthesized as their Na salts using the well-known azo-coupling method (Alsantali *et al.*, 2022 ▸; Kennedy *et al.*, 2001 ▸). Na salts were converted to NH_4_ or K salts by reaction with a slight excess of either NH_4_Cl or KCl in warm water. Solutions were filtered to give clear aqueous solutions and then allowed to evaporate for 2 to 7 d. This gave yellow or yellow–orange crystals of the desired salt forms that were suitable for single-crystal diffraction analysis. FT–IR spectra were measured as KBr discs using a Nicolet Avatar 380 spectrometer. Raman data were measured from solids using a Reinshaw Ramascope with excitation at 785 nm. Aqueous UV–Vis spectra were measured using a Cary 300 Bio spectrophotometer and solid-state UV–Vis spectra were measured using a Phillips PU8749 spectrophotometer. Measurements on K**1** were made at Station 9.8 of the Daresbury SRS. All other measurements were made using standard Rigaku Synergy-i (NH_4_ **4**) or Enraf–Nonius KappaCCD (all other structures) laboratory diffractometers equipped with CCD detectors.

### Spectroscopic data

NH_4_ **1**, FT–IR (cm ^−1^): 3421, 3267, 3088, 1598, 1501, 1280, 1193, 1040. Raman (cm^−1^): 1120, 1147, 1185, 1435, 1459, 1594. UV–Vis (λ_max_, nm): 348 (aq), 346 (solid).

NH_4_ **2**, FT–IR (cm ^−1^): 1629, 1588, 1419, 1337, 1198, 1116, 1034, 999, 840, 804. Raman (cm^−1^): 239, 317, 348, 446, 836, 1036, 1122, 1161, 1197, 1324, 1375, 1419, 1591. UV–Vis (λ_max_, nm): 417 (aq), 334 (solid).

NH_4_ **3**, FT–IR (cm ^−1^): 1634, 1603, 1501, 1398, 1383, 1275, 1193, 1116, 1034, 1004, 835. Raman (cm^−1^): peaks masked by fluorescence. UV–Vis (λ_max_, nm): 386 (aq), 332 (solid).

NH_4_ **4**, FT–IR (cm ^−1^): 1598, 1511, 1403, 1213, 1116, 1075, 1024, 844, 814, 691. Raman (cm^−1^): 1034, 1115, 1143, 1197, 1315, 1356, 1387, 1419, 1441, 1589. UV–Vis (λ_max_, nm): 459 (aq), 418 (solid).

NH_4_ **5**, FT–IR (cm ^−1^): 3405, 3190, 2366, 1597, 1495, 1423, 1177, 1136, 1034. Raman (cm^−1^): 1138, 1183, 1422, 1452 (somewhat masked by fluorescence). UV–Vis (λ_max_, nm): 348 (aq), 351 (solid).

K**1**, FT–IR (cm ^−1^): 1593, 1460, 1372, 1178, 850, 717. Raman (cm^−1^): 628, 794, 922, 1102, 1121, 1147, 1158, 1309, 1396, 1435, 1590, 1607. UV–Vis (λ_max_, nm): 348 (aq), 334 (solid).

K**2**, FT–IR (cm ^−1^): 3472, 3390, 1593, 1469, 1372, 1198, 1121, 1039, 839, 803. Raman (cm^−1^): 1033, 1122, 1161, 1198, 1326, 1374, 1418, 1592. UV–Vis (λ_max_, nm): 430 (aq), 336 (solid).

K**3**, FT–IR (cm ^−1^): 1644, 1460, 1372, 1193, 1116, 1029, 839, 716. Raman (cm^−1^): 780, 921, 1036, 1120, 1152, 1427, 1588. UV–Vis (λ_max_, nm): 387 (aq), 345 (solid).

K**4**, FT–IR (cm ^−1^): 1597, 1515, 1382, 1351, 1316, 1259, 1223, 1182, 1116, 1049, 1003. Raman (cm^−1^): 739, 1032, 1117, 1143, 1195, 1315, 1355, 1392, 1421, 1440. UV–Vis (λ_max_, nm): 458 (aq), 413 (solid).

K**5**, FT–IR (cm ^−1^): 3390, 3078, 1603, 1501, 1383, 1188, 1034, 845. Raman (cm^−1^): 1136, 1169, 1185, 1424, 1454 (somewhat masked by fluorescence). UV–Vis (λ_max_, nm): 347 (aq), 348 (solid).

### Refinement

Crystal data, data collection and structure refinement details are summarized in Table 1 ▸. For K**4**, the azo­benzene core of one of the two crystallographically independent dye anions was treated as disordered over two sites. In a similar way, one of the four independent SO_3_ groups of K**3** was also treated as disordered over two sites, a rotation about the C—S bond giving alternative positions for the three O atoms. In both cases, appropriate restraints and constraints on the displacement parameters were added so as to ensure that the structures approximated normal behaviour. Finally, one water mol­ecule of NH_4_ **4** was modelled as rotationally disordered about atom O2*W* so that the water mol­ecule has three independent H-atom sites. Where possible, H atoms attached to O or to N atoms were positioned as found in difference syntheses and refined freely and isotropically. Where riding models were required, *X*—H bond lengths were set at 0.88 (1) Å. All H atoms bound to C atoms were included in riding models, with C—H = 0.95 or 0.99 Å for CH and CH_2_ groups, respectively. For all H atoms in riding models, *U*_iso_(H) values were set to 1.2*U*_eq_ of the parent atom.

## Results and discussion

**Figure 1:**
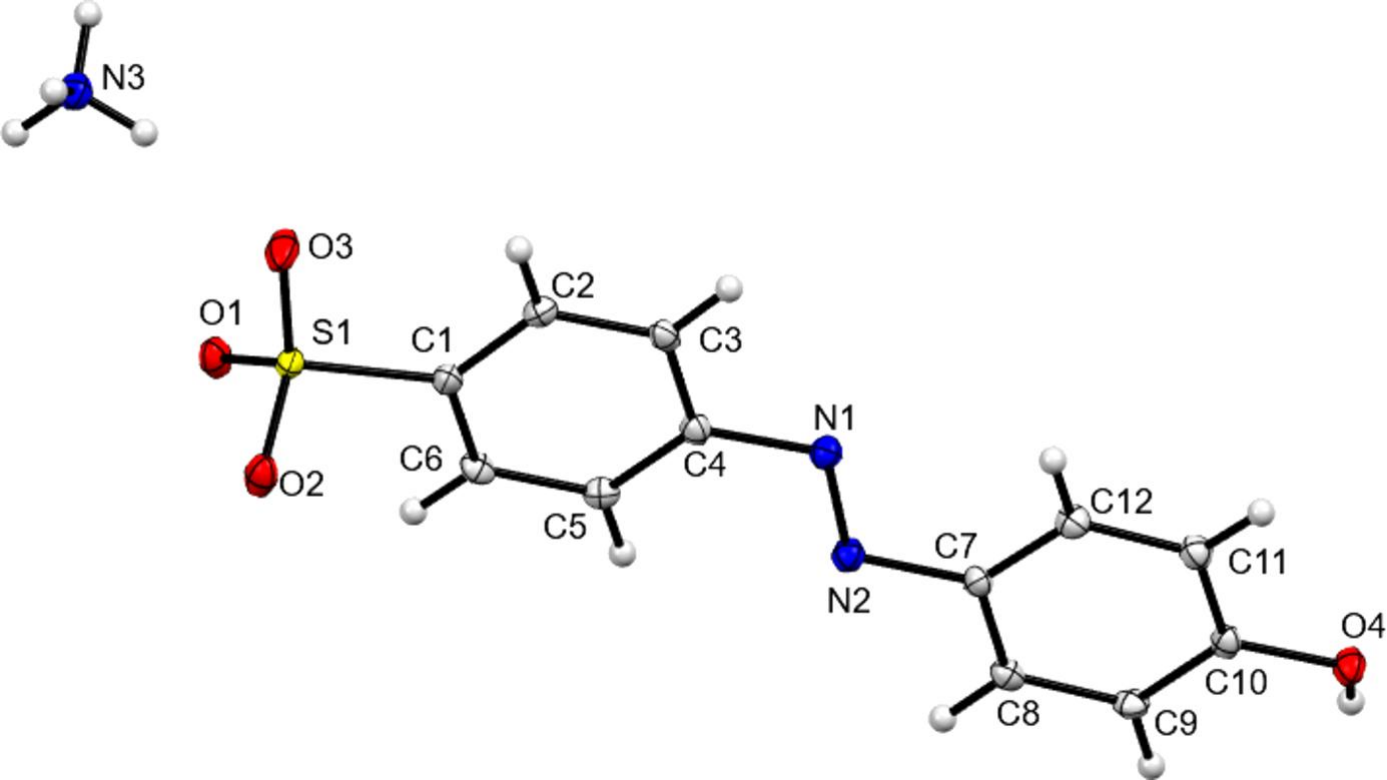
The asymmetric unit contents of NH_4_ **1**. Here and elsewhere displacement ellipsoids are drawn at the 50% probability level and H atoms are drawn as small spheres of arbitrary size.

**Figure 2:**
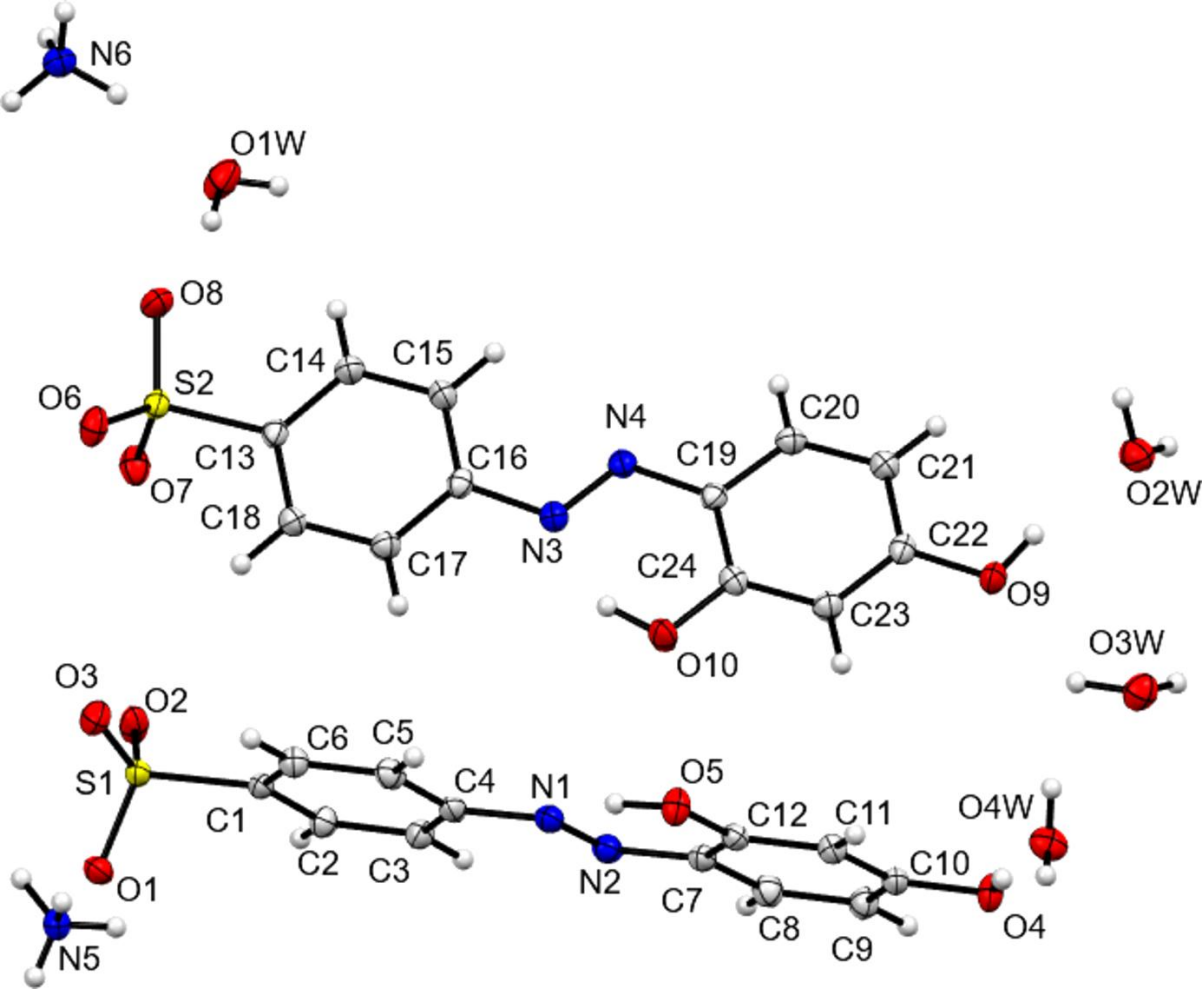
The asymmetric unit contents of NH_4_ **2**.

**Figure 3:**
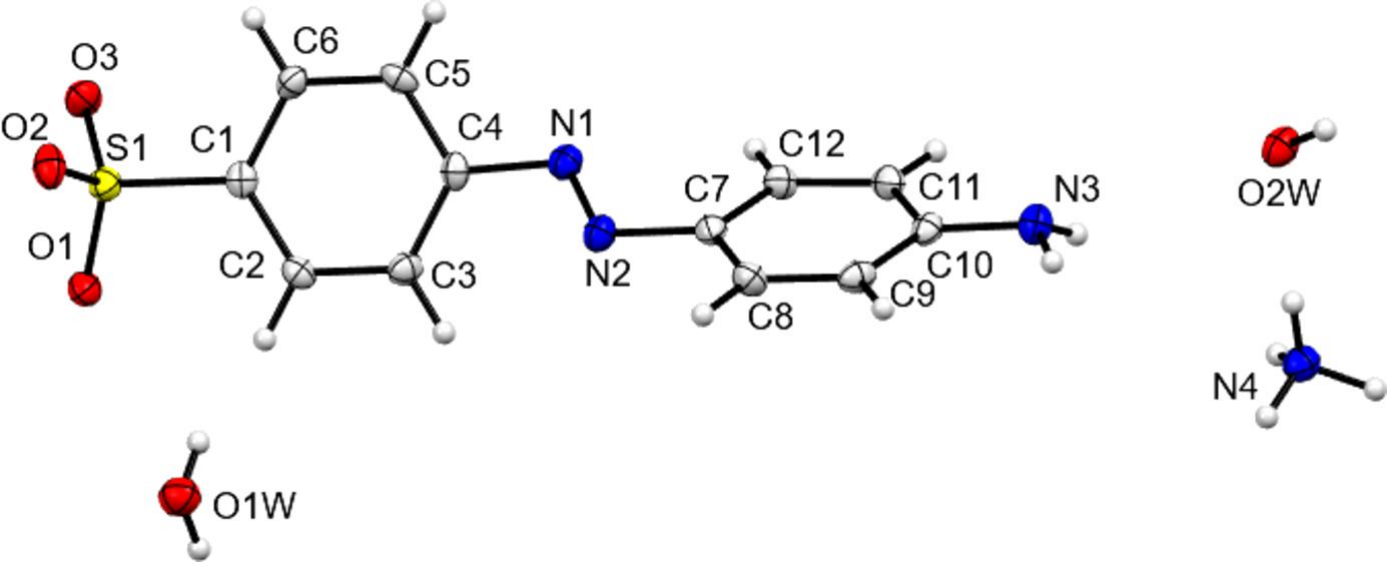
The asymmetric unit contents of NH_4_ **3**. A twofold rotation axis passes through atom O2*W*.

**Figure 4:**
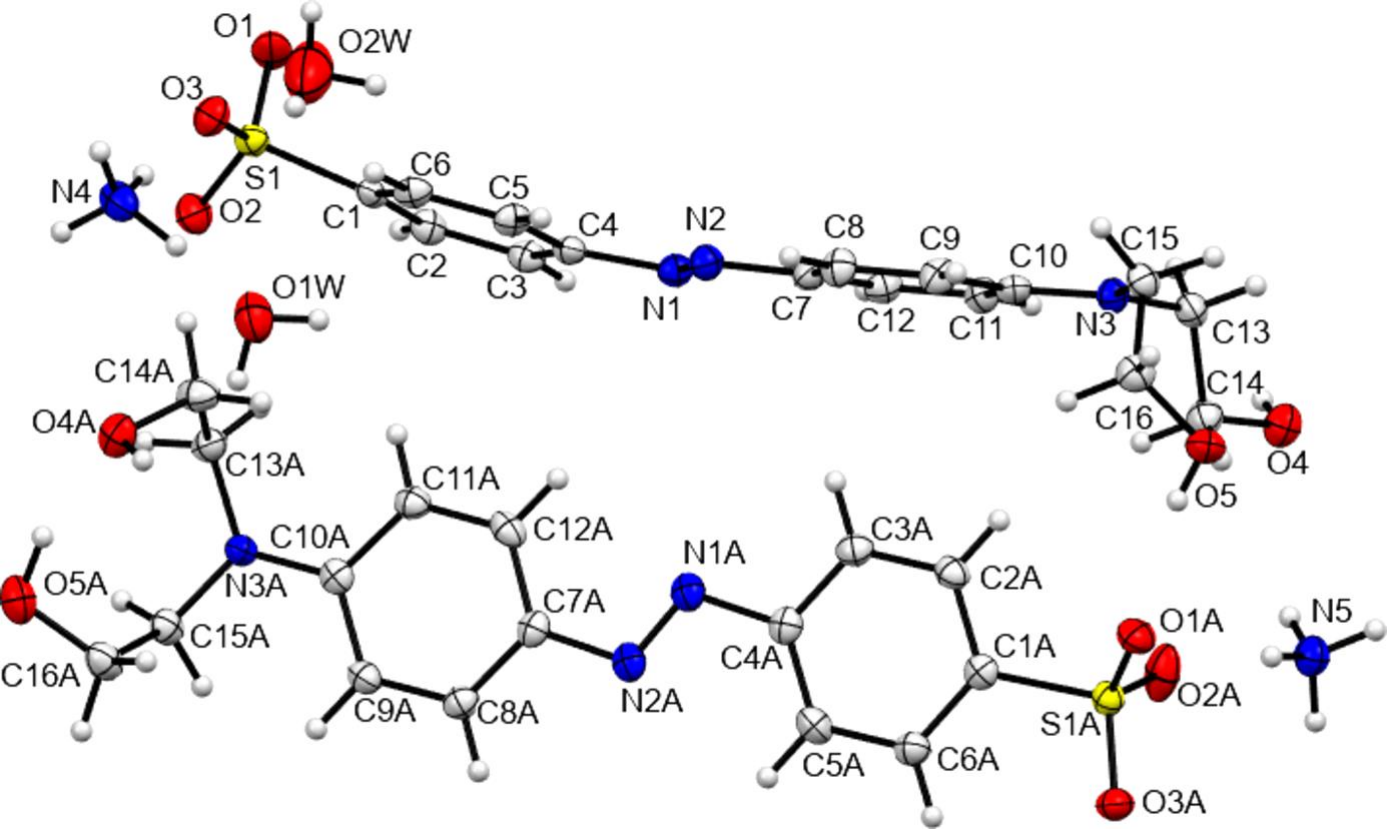
The asymmetric unit contents of NH_4_ **4**. The water mol­ecule labelled O2*W* is disordered so as to give three independent H-atom sites.

**Figure 5:**
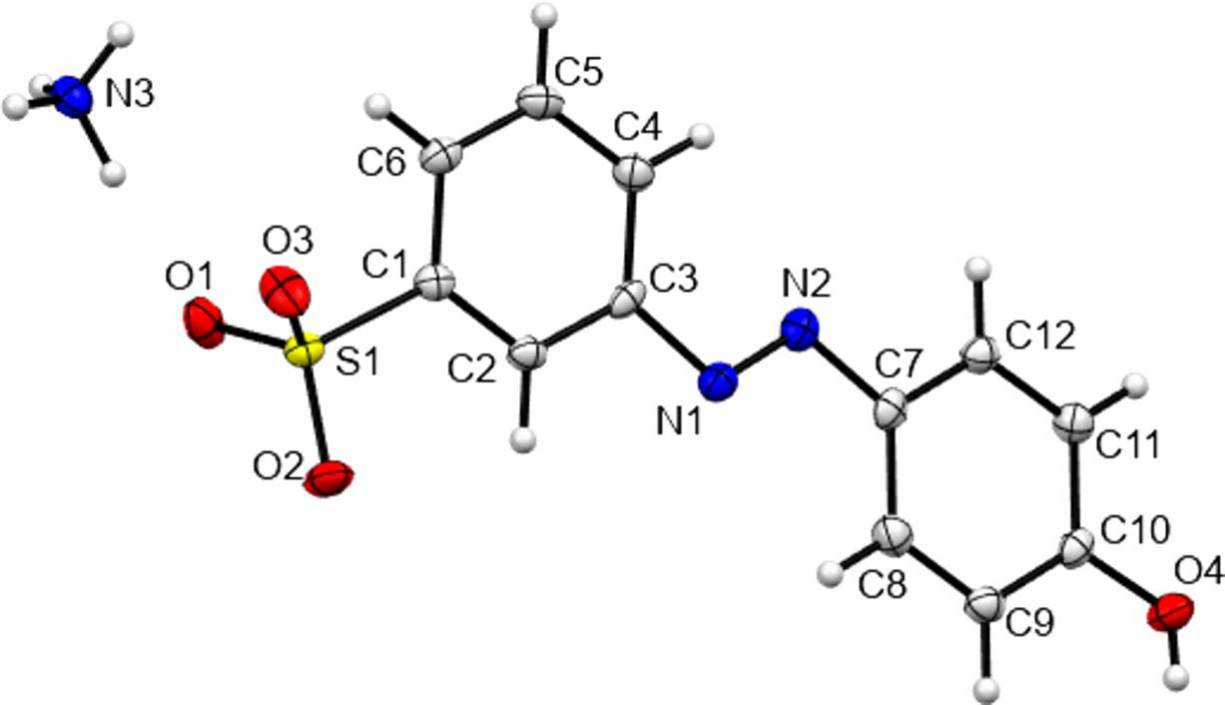
The asymmetric unit contents of NH_4_ **5**.

**Figure 6:**
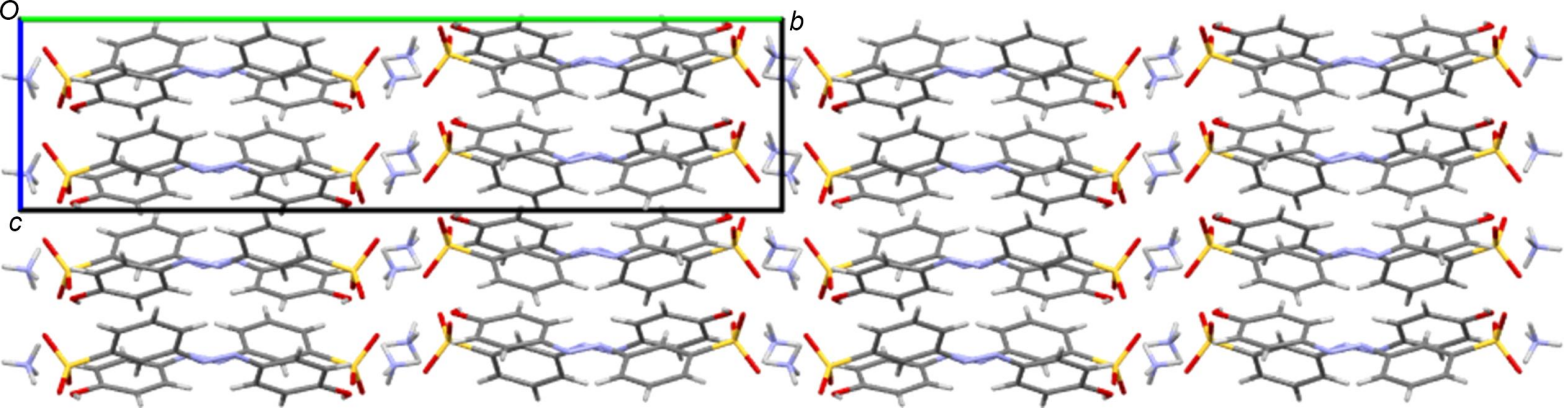
Packed structure of NH_4_ **5** viewed along the *a* axis. Note the simple layering structure with alternating hydro­phobic (aryl­azo) and hydro­philic (cation) layers parallel to the *ac* plane.

All structures discussed are of crystal samples obtained from aqueous recrystallizations. Aqueous conditions were chosen to best reflect normal synthesis and usage of sulfonated azo dyes. It is possible to dry the hydrate structures described to give anhydrous materials. However, no polymorph screen has been attempted and so the existence of other forms is entirely likely. Selected crystallographic and refinement parameters are given in Table 1 ▸ and selected geometric parameters are given in Tables 2 to 14 ▸ ▸ ▸ ▸ ▸ ▸ ▸ ▸ ▸ ▸ ▸ ▸ ▸. Representations of the crystal structures of the ammonium salt forms NH_4_ **1**, NH_4_ **2**, NH_4_ **3**, NH_4_ **4** and NH_4_ **5** are given in Figs. 1 to 5 ▸ ▸ ▸ ▸ ▸. Both the phenol derivatives NH_4_ **1** and NH_4_ **5** are simple anhydrous salts with an NH_4_^+^ cation and an azo anion in the asymmetric unit. The other species are all hydrated forms as shown in Table 15 ▸. Additionally, both NH_4_ **2** and NH_4_ **4** are *Z*′ = 2 structures with two cation/anion pairs per asymmetric unit, as well as their accompanying water mol­ecules. The bond lengths about the azo chromophores are in good agreement with those described for *s*-block metal salts of similar azo anions (Kennedy *et al.*, 2001 ▸, 2020 ▸). The N=N bonds range from 1.254 (3) to 1.275 (2) Å, whilst the C—N bonds show ranges of 1.423 (2)–1.439 (2) and 1.396 (2)–1.425 (3) Å for the bonds involving the C atoms of the sulfonated and nonsulfonated rings, respectively. The generally shorter bonds of the latter are due to resonance with the OH and amine substituents. It is noteworthy that it is NH_4_ **2** that displays both the longest N=N bond and the shortest N—C bond. The intra­molecular O—H⋯N hydrogen bonding of NH_4_ **2** raises the possibility of tautomerism in this species and, although the electron density shows that the compound clearly exists largely as the azo tautomer, the bond lengths observed tend to reflect a small contribution from the alternative hydrazone tautomer. [See Kennedy *et al.* (2020 ▸) for a detailed discussion of factors that influence bond lengths and hence colour in similar azo dyes, and Yatsenko *et al.* (2024 ▸) for a discussion on azo/hydrazone tautomerization in solid-state aryl­azo compounds.] Most of the azo anions have planar conformations, with angles between the planes of the aromatic rings ranging from 2.23 (9) to 12.32 (11)°. Despite this, the azo group does form a small step between the two parallel ring planes [*e.g.* in NH_4_ **1**, atom N2 lies 0.473 (5) Å out of the plane defined by atoms C1–C6]. The exception is the anion of NH_4_ **3**, which adopts a twisted conformation with an angle of 58.76 (11)° between the planes of its two aromatic rings. This out-of-plane twist in the solid state should alter the resonance through the azo­benzene fragment and may contribute to the relatively large difference found between the solution-state and solid-state λ_max_ values (386 *versus* 332 nm). It has previously been shown for *s*-block metal salt forms that the planar or twisted conformation of *ortho*-sulfonated azo anions correlates with the packing motifs observed. Thus, twisted azo species gave structures with a simple alternating layer structure, *i.e.* layers of organic anions alternating with hydro­philic layers containing the cations and water mol­ecules. In contrast, planar azo anions gave structures with organic bilayers (Kennedy *et al.*, 2009 ▸). This is not observed here; all the ammonium salts of the *para*- and *meta*-sulfonated anions **1** to **5** give simple layering structures with no bilayers, irrespective of the planarity of the anion (see Figs. 6 ▸ and 7 ▸ for examples).

Each H atom of every NH_4_^+^ cation acts as a hydrogen-bond donor to at least one O atom. There are no NH_4_-to-N hydrogen-bonding inter­actions. From Tables 2 ▸ to 6 and the summary Table 15 ▸, it can be seen that of the 28 independent N—H donors of the ammonium groups, 10 form bifurcated hydrogen bonds and two (atoms H8N of NH_4_ **2** and H1N of NH_4_ **5**) inter­act with three separate O-atom acceptors. Most ammonium cations inter­act with six O atoms, but one (that containing atom N4 of structure NH_4_ **4**) has a coordination number of 5 and one (containing atom N3 of NH_4_ **5**) has a coordination number of 7. In all five structures, the majority of the ammonium cation hydrogen-bond inter­actions are with the O atoms of the formally negatively charged SO_3_ groups. For the anhydrous species NH_4_ **1** and NH_4_ **5**, the ammonium ions also donate hydrogen bonds to the phenol OH groups; thus, hydrogen bonding from ammonium to both the SO_3_ head and the OH tail of the azo anions leads to the anions bridging between the inorganic/hydro­philic layers of the packing structures (Fig. 6 ▸). The hydrates NH_4_ **2** and NH_4_ **3** have no direct ammonium-to-tail-group hydrogen-bond inter­actions; instead, the water mol­ecules act as inter­mediaries or bridges and accept/donate hydrogen bonds from both ammonium and tail groups. Thus, for these hydrate species, inter­actions between the hydro­phobic and hydro­philic layers of the packing structure is *via* the water mol­ecules. Despite featuring a lower cation coordination number, in hydrate NH_4_ **4**, the cations donate hydrogen bonds to all of the different types of acceptor groups available, *i.e.* to the SO_3_ heads, to the OH groups of the tail and to bridging water mol­ecules.

**Figure 8:**
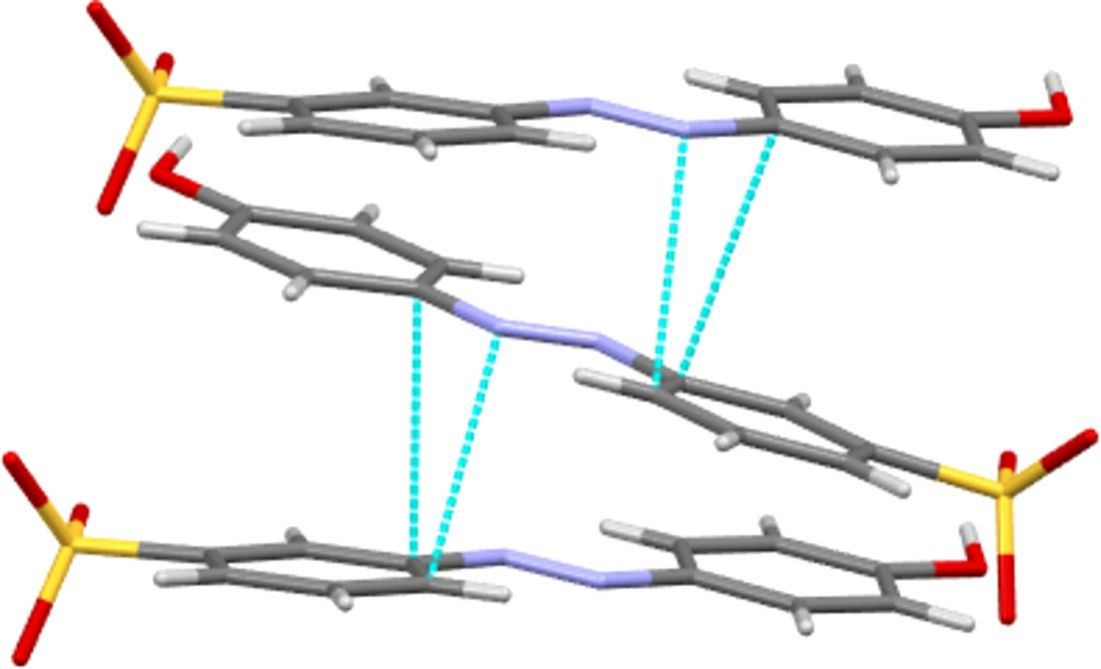
Part of azo stack propagating parallel to the *c* axis in the structure of NH_4_ **5**.

**Figure 9:**
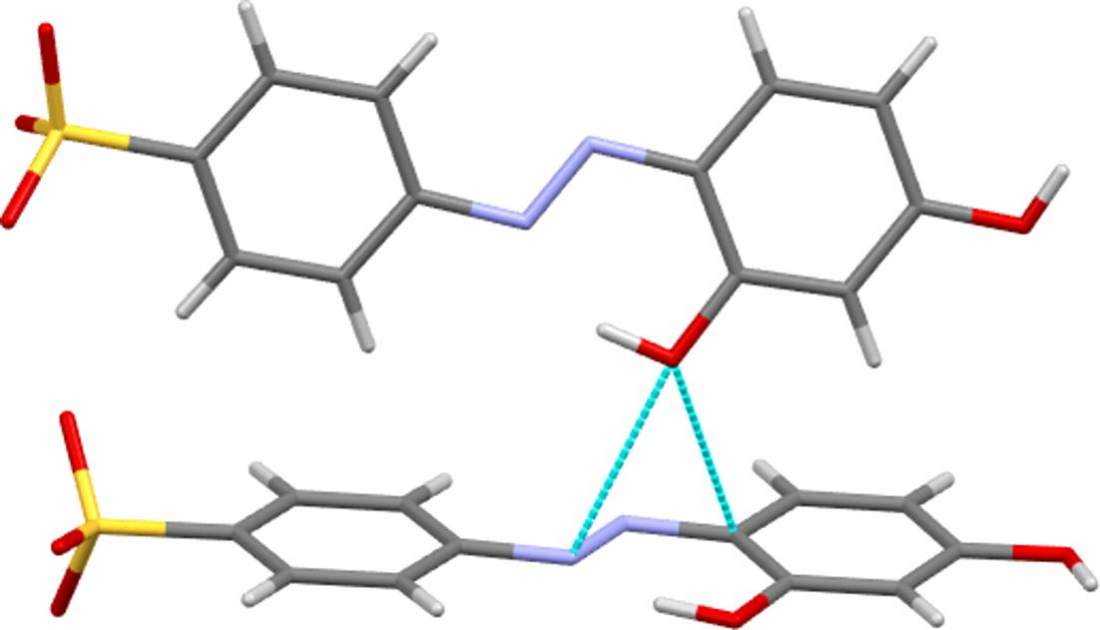
Dimeric close contact between the two azo anions of the asymmetric unit of NH_4_ **2**.

As they are situated at the centre of the hydro­phobic layers, none of the –N=N– chromophore units take part in inter­molecular hydrogen bonding. Indeed, the azo groups of NH_4_ **1**, NH_4_ **3** and NH_4_ **4** have no inter­molecular contacts less than the sum of the van der Waals radii. NH_4_ **5** is the only species of the five to show face-to-face π-contacts between the azo­benzene units, with closest N2⋯C4 and C7⋯C3 contact distances of 3.160 (4) and 3.352 (4) Å, respectively. This forms the stacking motif seen extending parallel to the *c* axis in Fig. 8 ▸. In NH_4_ **2**, the *ortho*-OH substituent of one of the independent azo anions of the asymmetric unit approaches the azo group of the other anion [O10⋯N1 = 3.052 (2) Å], creating dimeric pairs of anions (Fig. 9 ▸).

**Figure 10:**
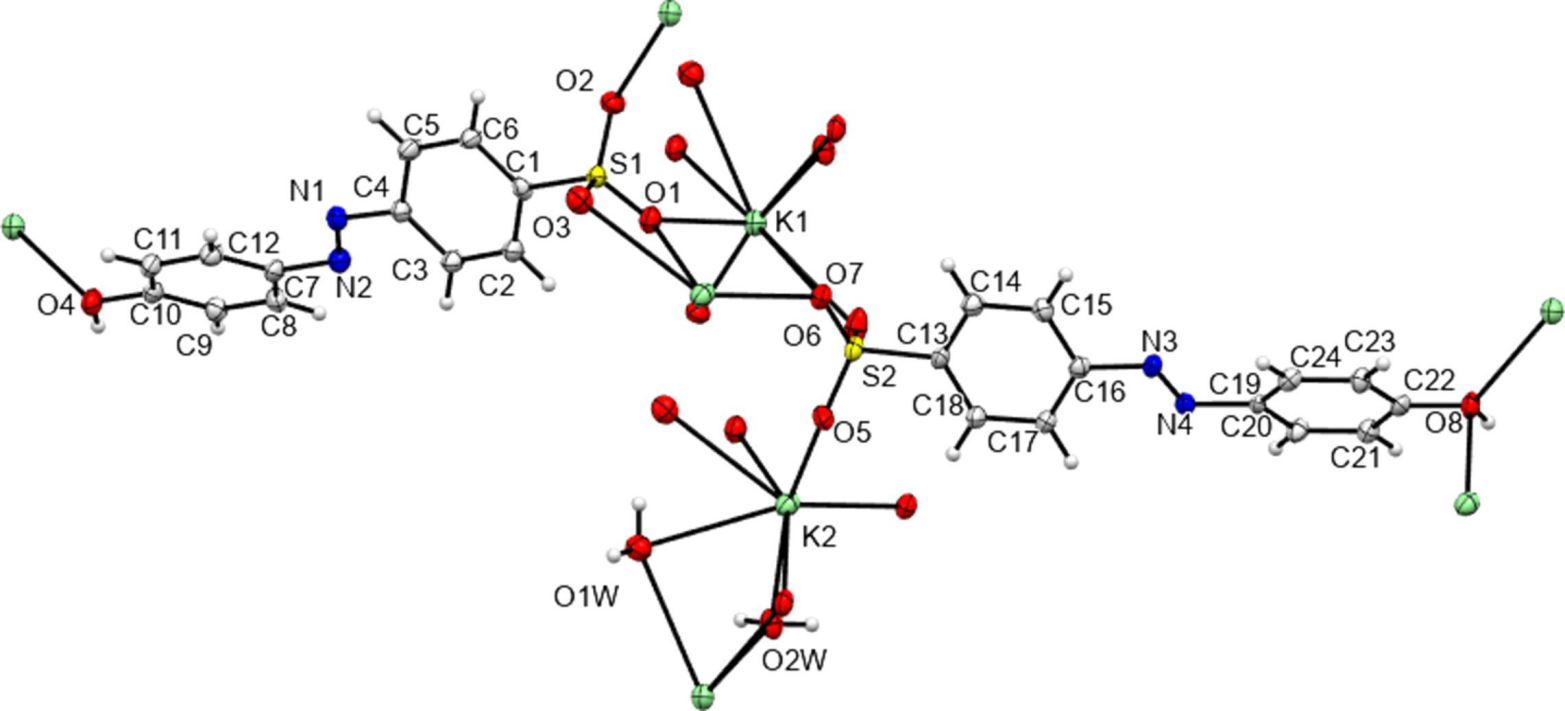
The contents of the asymmetric unit of K**1** extended so as to show the coordination geometry about each independent K centre.

**Figure 11:**
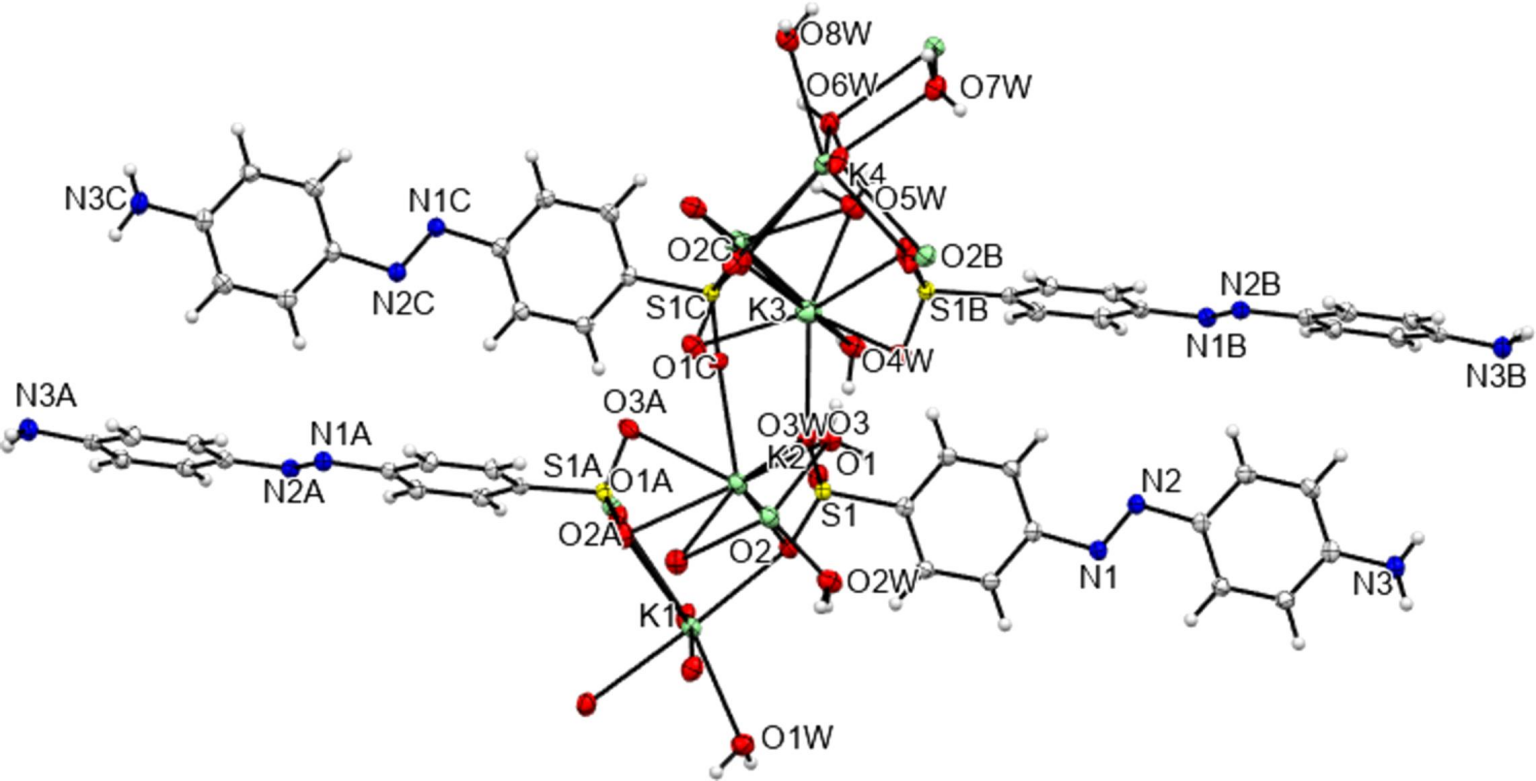
The contents of the asymmetric unit of K**3** extended so as to show the coordination geometry about each independent K centre. Disorder in the SO_3_ group of atom S1*C* is not shown for clarity.

**Figure 12:**
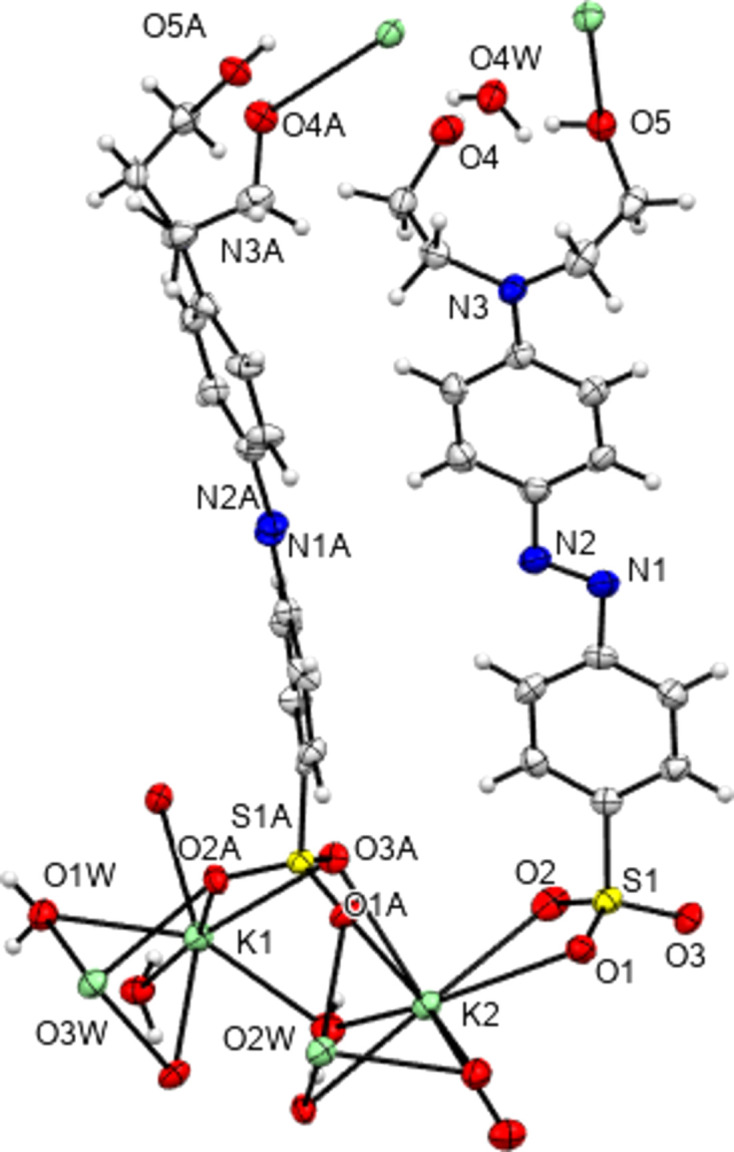
The contents of the asymmetric unit of K**4** extended so as to show the coordination geometry about each independent K centre. Disorder in the azo ion of atom N1*A* is not shown for clarity.

**Figure 13:**
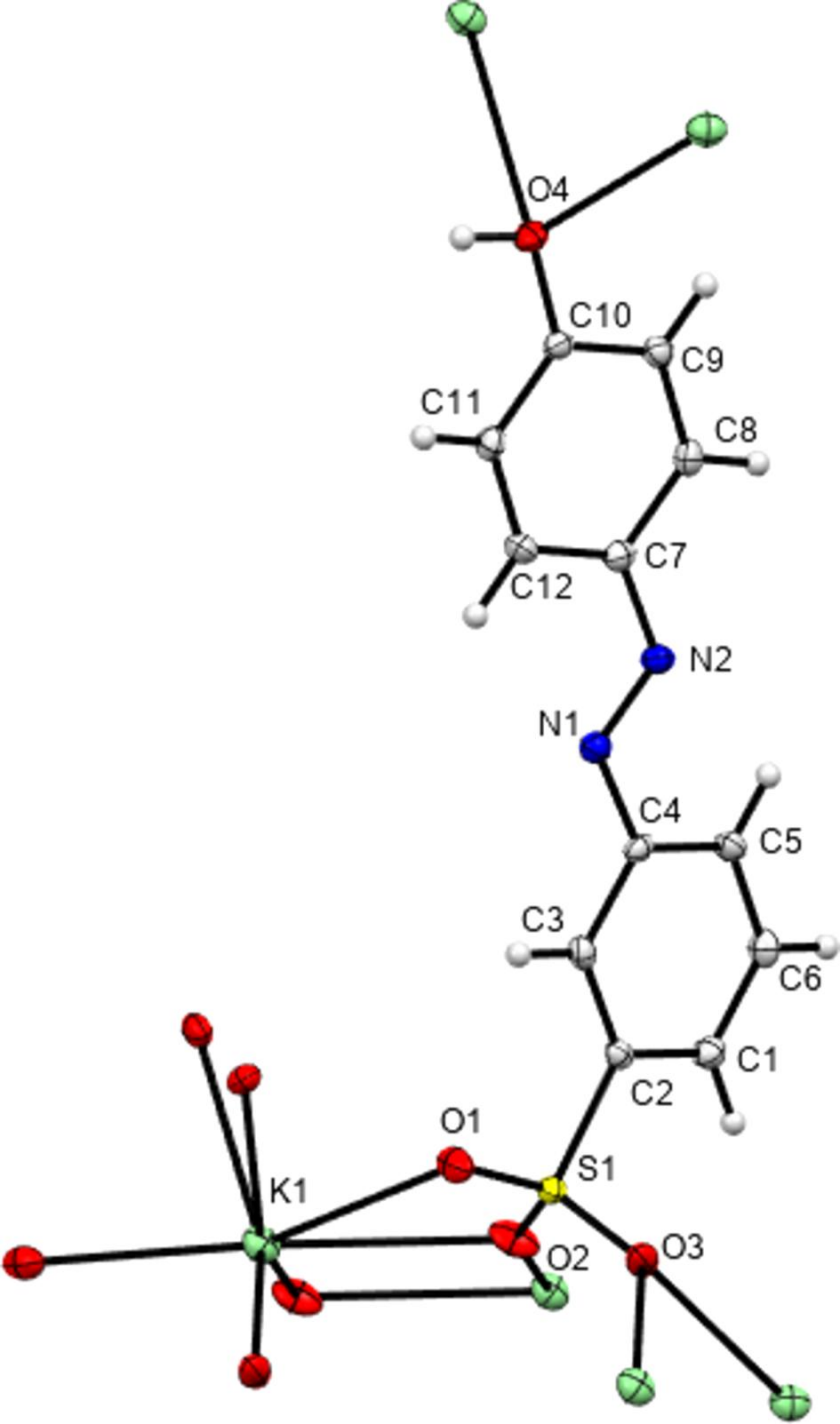
The contents of the asymmetric unit of K**5** extended so as to show the coordination geometry about the K centre.

**Figure 14:**
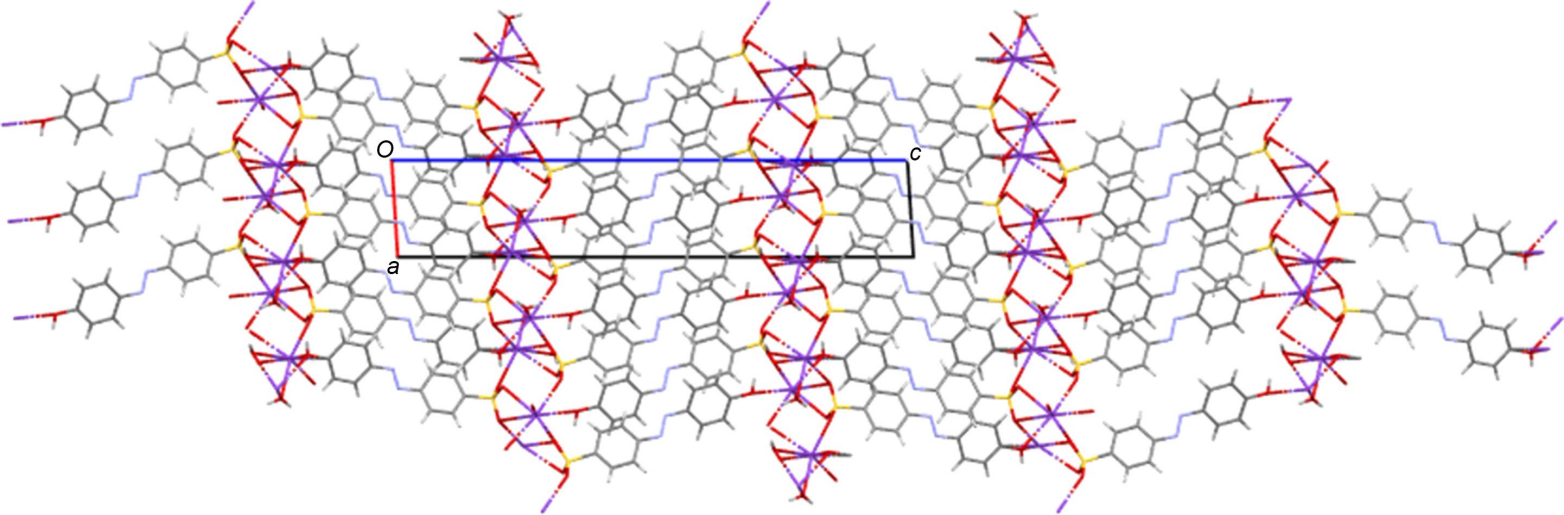
Packing diagram of K**1** as viewed along the *b* axis. Note the alternating hydro­phobic and hydro­philic layers that propagate parallel to the *ab* plane. The three-dimensional coordination polymer propagates parallel to *a* and *b* (and throughout the hydro­philic layers) *via* sulfonate and water bridges between the K centres. The coordination polymer propagates parallel to *c via* K bonding to both the sulfonate head and the OH tail of the azo anions.

The structure of K**2** was reported by Kennedy *et al.* (2004 ▸). Here, we report the remaining K-salt structures of azo anions **1** to **5**. Figs. 9 to 13 ▸ ▸ ▸ ▸ ▸ show the fundamental features of these structures and selected geometric parameters are given in Table 7 ▸ to 14. K**5** is anhydrous and a *Z*′ = 1 structure, but all other K salts were isolated as hydrates and have *Z*′ = 2 (or 4 for K**3**); see Table 15 ▸ for a summary of these and other structural features. Values for the disordered anion of K**4** have been excluded from the geometric discussion below. The azo N=N bond lengths range from 1.258 (3) to 1.276 (3) Å, with the long value again found for the salt of **2** with its intra­molecular O—H⋯N hydrogen bond and the possibility of tautomerization. The C—N bonds range from 1.420 (3) to 1.442 (4) and from 1.397 (3) to 1.432 (4) Å for the bonds to the sulfonated heads and to the phenol/amine tails, respectively. Again, it is K**2** that presents the most different bond lengths. All these bond lengths are similar to those found for the NH_4_ salts, above, and for related sulfonated azo salt forms (Kennedy *et al.*, 2020 ▸). Most azo anions have planar geometries [angles between ring planes = 0.93 (7) to 8.15 (22)°], with the exception of K**1**, where the two independent azo anions are both twisted [angles between ring planes = 43.15 (7) and 40.48 (8)°]. This contrasts with the ammonium salt structures, where all species were planar except for the twisted NH_4_ **3**. As with the NH_4_ species, all five K structures both planar and twisted have simple layered packing systems with layers of hydro­philic/inorganic species (K and water) alternating with layers of a hydro­phobic/organic nature (azo­benzene) (see Fig. 14 ▸ for an example).

**Figure 15:**
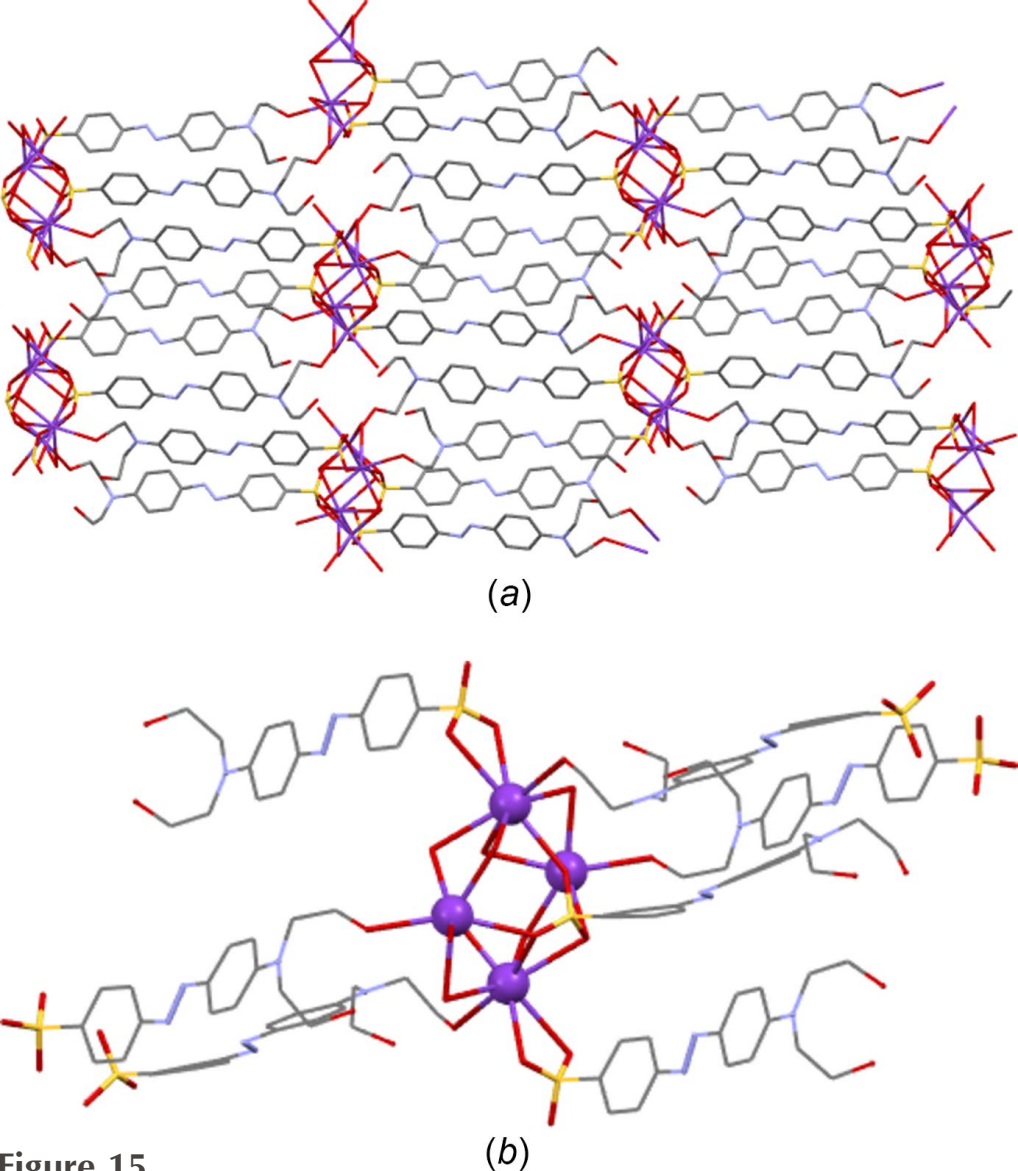
(*a*) View of K**4** illustrating that here K tetra­mers link into a two-dimensional coordination polymer only *via* through-azo inter­actions with the head and tail groups of the azo anions. (*b*) Detail of one K_4_ tetra­meric unit.

**Figure 16:**
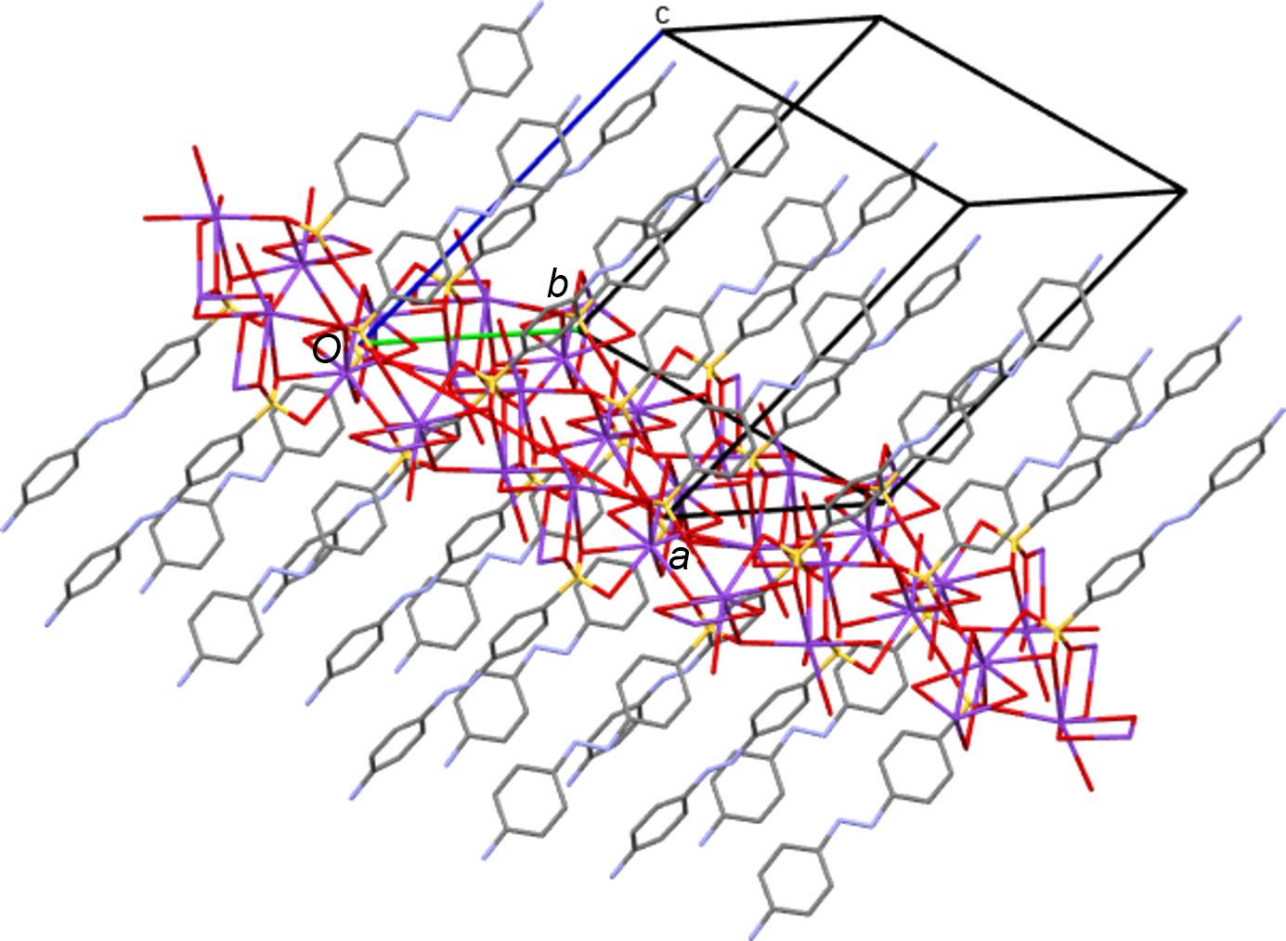
The two-dimensional coordination polymer of K**3** propagates only *via* sulfonate groups and water groups that bridge between K centres – there is no head-to-tail through-azo bridge.

**Figure 17:**
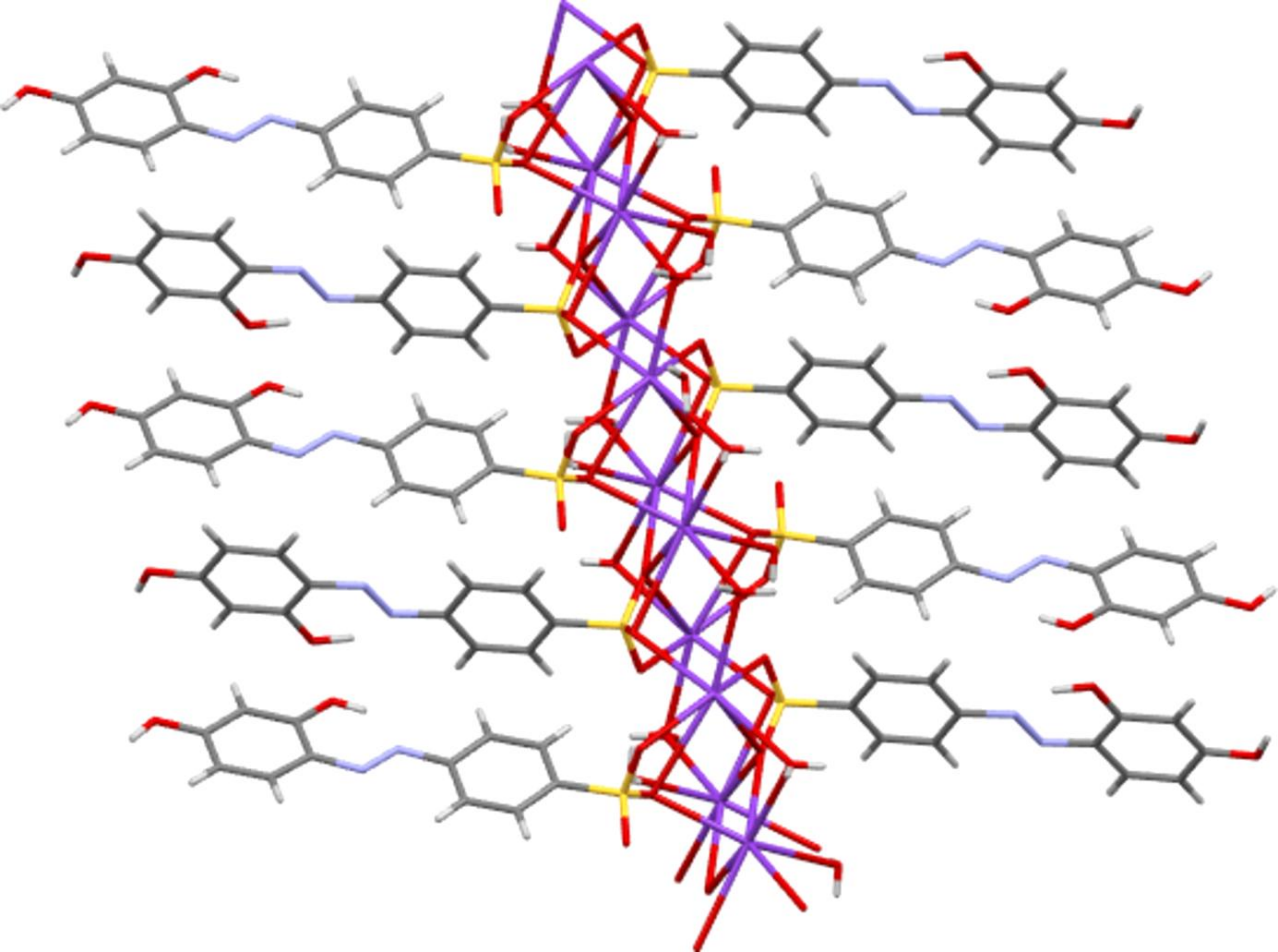
The one-dimensional coordination polymer structure of K**2**, with the coordination chain propagating parallel to the *a* direction (Kennedy *et al.*, 2004 ▸).

Each K centre has a coordination number of 7 or 8 (see Table 15 ▸), with all bonds from K being to O atoms. All K centres inter­act with SO_3_ groups and, where present, with water mol­ecules. In K**1**, K**4** and the anhydrous K**5**, there are also bonds formed between K and the OH groups of the tails of the azo anions. No such K-to-tail bonds are formed to the OH groups of K**2** or to the NH_2_ groups of K**3**, instead these form only hydrogen bonds (Table 10 ▸). This throws up a similarity to the NH_4_ structures, where no direct NH_4_-to-tail hydrogen bond was found for NH_4_ **2** or NH_4_ **3**, despite such bonds existing in all other compounds. As would be expected from the positions of the sulfonate groups and the nature of the cation, all five potassium salts form structures described elsewhere as class three ‘higher connectivity’ azo colourant structures (Kennedy *et al.*, 2004 ▸). Structures K**1** and K**5** both form three-dimensional coordination polymers, with K—O bonds (K-to-SO_3_ in K**5** and both K-to-SO_3_ and K-to-OH_2_ in K**1**) forming bridges between K centres and allowing propagation of the polymer in two dimensions, whilst the third dimension features bridging between K centres through the body of the azo ion and hence utilizing both the head and tail functional groups of the azo anions (Fig. 14 ▸). Despite also featuring bridges between K centres *via* head-to-tail contacts with the azo anion, K**4** is only a two-dimensional coordination polymer. Here, SO_3_ and OH_2_ bridges between K centres lead only to discrete K_4_ tetra­mers and it is only the through-azo head-to-tail inter­actions that allow the coordination polymer to propagate parallel to the *b* and *c* directions (Fig. 15 ▸). With no inter­action with the amine tail of the azo anion, K**3** displays a two-dimensional coordination polymer structure based solely on SO_3_ and OH_2_ bridges between K centres. Here, the polymeric structure propagates parallel to the *a* and *b* directions (Fig. 16 ▸). K**2** also does not feature any K-to-tail inter­actions and here only a one-dimensional coordination poly­mer is formed. SO_3_ and OH_2_ bridges between K centres leads to a polymer that propagates parallel to the crystallographic *a* direction (Fig. 17 ▸).

The structures of the five Na salt equivalents of **1** to **5** crystallized from water solutions are all available from the literature (Kennedy *et al.*, 2001 ▸, 2020 ▸; Dodds *et al.*, 2017 ▸). As summarized in Table 15 ▸, Na**1** forms a one-dimensional coordination polymer, whilst the other four Na structures all form two-dimensional coordination polymers. All Na centres are six-coordinate, except for those in Na**4**, which are seven-coordinate. All Na centres bond to O atoms of the sulfonate groups and to water ligands. Na**1** and Na**2** do not form bonds from Na to the tail phenol groups, but the other three structures do form Na-to-tail bonds to N or to O atoms. The azo anions that form bonds to Na with their tail groups are thus different from those that inter­act with K or with NH_4_, as described above. The generally lower coordination numbers of the Na salts (6 or 7) as compared to the K salts (7 or 8) seems to be reflected in a similarly generally lower dimensionality of coordination polymers (one- or two-dimensional for Na, and one- to three-dimensional for K), but this is not a hard and fast rule. This is shown by comparing Na**2** with K**2**, where Na has a coordination number of 6 *versus* 7 for K, but where the Na compound is a two-dimensional coordination polymer and the K compound only one-dimensional.

Comparing the unit-cell dimensions given in Table 15 ▸, it can clearly be seen that there are two isostructural pairs. The space group and unit-cell dimensions of NH_4_ **2** match those of K**2** and there is a similar match between NH_4_ **5** and K**5**. Based on the unit-cell dimensions, none of the Na species are isostructural with any NH_4_ or K salt form. For the NH_4_ **5** and K**5** pair, all generalized structural descriptors (such as *Z*′, hydration state and the coordination number of the cation) are identical, as would be expected for a truly isostructural pair. However, the descriptors given for the NH_4_ **2** and K**2** pair in Table 15 ▸, whilst mostly identical, do differ with respect to the coordination number of the cation. The NH_4_ ions are given as making six inter­actions each, whilst the K centres are given as each making seven inter­actions. Investigation shows that this difference is not just a case of an inappropriate cut-off distance being used to calculate potential hydrogen-bonding inter­actions. For example, the ‘extra’ seventh bond for atom K1 is an inter­action with a sulfonate O atom. The closest sulfonate O atom to the equivalent ammonium ion in NH_4_ **2**, that has not already been accounted for as a hydrogen-bonding inter­action, gives an N⋯O distance of over 4.2 Å, which is clearly too long for a hydrogen bond; see Table 3 ▸ for genuine N⋯O hydrogen-bond distances. Thus, although NH_4_ **2** and K**2** have similar unit-cell dimensions and similar compositions, small changes in the orientations of the sulfonate groups and the cations allow the ammonium and potassium cations to make somewhat different inter­actions from each other. The ability of isostructural structures to tolerate small changes within a given packing motif has been discussed recently by Bombicz (2024 ▸).

Expanding from the 15 structures of Table 15 ▸ to other literature structures, of the five azo anions studied here, only **3** has had the structure of its Rb salt form reported (Kennedy *et al.*, 2004 ▸). The structure of Rb**3** is found to be isostructural with that of NH_4_ **3** (compare *C*2/*c*, 35.256, 7.738, 10.674 Å and 99.88° with the values given for NH_4_ **3** in Table 1 ▸). As with NH_4_ **2** and K**2** above, all structural descriptors match, except for the Rb cation having a higher coordination number than the ammonium cation (8 *versus* 6). Other sulfonated monoazo NH_4_ salt form structures available are those of di­ammonium Orange G tetra­hydrate and of the nitrile-substituted [NH_4_][O_3_S(C_6_H_4_)NN(C_6_H_4_)NHCH_2_CH_2_CN]·H_2_O (Ojala *et al.*, 1994 ▸; Astbury *et al.*, 2013 ▸). For these azo anions, the only available comparisons for the ammonium salts are the Na and mixed Na/K and Na/Rb salt forms of Orange G and the Na salt of the nitrile (Kennedy *et al.*, 2006 ▸; Astbury *et al.*, 2013 ▸). Based on unit-cell dimensions, the nitrile shows no isostructurality. There are however similarities, if not exact matches, between the unit cells of the Orange G structures of Table 16 ▸; see com­ments below for a fuller discussion of these features.

**Figure 18:**
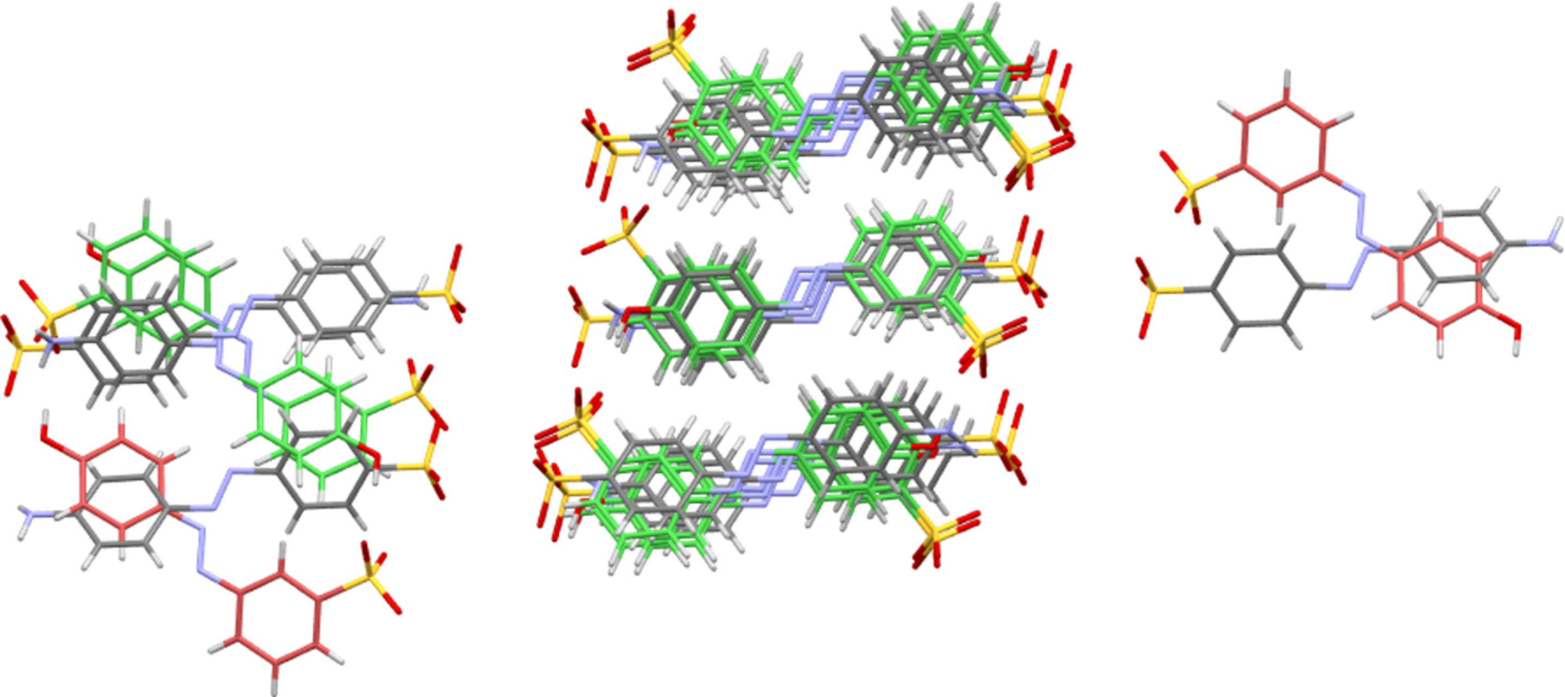
15-fragment overlay of the structure of NH_4_ **3** (multicoloured) with that of BAHNIK (Ag**5**, green and red; Dodds *et al.*, 2017 ▸). The anionic azo fragments in the central layer give reasonable packing matches for the azo­benzene units, but the anions of the neighbouring layers do not match well.

The ‘crystal packing similarity’ tool available within *Mercury* (Macrae *et al.*, 2020 ▸) can be used to find packing simi­larity regardless of any matches in the unit-cell dimension. For instance, it has been used to find similarities in the packing behaviour of dopamine and tyramine fragments in the salt and hydrate forms of these active pharmaceutical ingredients (APIs), regardless of counter-ions, solvents and the identity and protonation state of the phenyl­ethyl­amine fragment itself (Kennedy *et al.*, 2023 ▸). This tool was applied to the 15 structures of Table 15 ▸ and to the other relevant NH_4_, Na, K, Rb, Cs and Ag salt structures found in the Cambridge Structural Database (CSD; Groom *et al.*, 2016 ▸). The packing analysis was set up to examine packed fragments consisting of 15 sulfonated azo mol­ecular units. Other components (cations and solvent mol­ecules) were ignored. Indeed, in order to simplify the definition of a ‘mol­ecular fragment’, all metal ions were deleted from the CIF files used for these analyses. Only the three pairs previously identified as being isostructural from their unit-cell dimensions (*i.e.* NH_4_ **2** and K**2**, NH_4_ **5** and K**5**, and NH_4_ **3** and Rb**3**) had azo anion packing that matched at a 15/15 level with a 20% tolerance allowed. However, it was found that NH_4_ **3** also matched at a 13/15 level with the isostructural pair of structures having CSD refcodes BAH­NAC and BAHNIK (Dodds *et al.*, 2017 ▸). Surprisingly, these two structures are not forms of the *para*-NH_2_-substituted anion **3**, but correspond to the Na and Ag salt forms of the *meta*-OH-substituted anion **5**. Further investigation of these structures reveals that it is their layered natures that lead to this partial match. Those azo fragments that lie within a single hydro­phobic layer of NH_4_ **3** match well with their equivalents in Na**5** and Ag**5**, despite the differences in SO_3_ position and the different chemical natures of the NH_2_/OH tail groups. However, the azo fragments within the neighbouring hydro­phobic layers do not match, being rotated with respect to one another (Fig. 18 ▸). A similar situation was found for the Orange G salts of Table 16 ▸. As is perhaps suggested by the differences in the unit-cell parameters, none of the metal salt forms were truly isostructural with the ammonium salt of Orange G. However, both the mixed Na/K and Na/Rb species matched the ammonium salt at a 12/15 level and the pure Na species matched at a 9/15 level. As with the case for **3** above, the matches within one organic layer are reasonable, but the structures do not match well when neighbouring layers are considered. Finally, examining the structures of Table 15 ▸ it can be seen that NH_4_ **1** and K**1** have somewhat similar *a* and *b* dimensions, whilst *c* is approximately doubled. Despite this, investigations showed that these structures did not show any significant similarity in the packing for the azo anions.

## Summary

Five new structures of ammonium salt forms of sulfonated azo dyes have been presented and compared to the equivalent Na and K salt structures. Despite being based on ammonium-to-sulfonate hydrogen bonds rather than metal-ion-to-sulfonate bonds, the ammonium salt forms are found to have structural types that are in many ways similar to those found for heavier alkali-metal ions (Na, K and Rb). The ammonium structures have the same simple alternating hydro­phobic/hydro­philic layer structures described earlier for *s*-block metal salt forms of *para*- and *meta*-sulfonated azo dyes (Kennedy *et al.*, 2004 ▸) and do not show the more complicated layering motifs seen elsewhere (Kennedy *et al.*, 2009 ▸). Two of the five ammonium structures (NH_4_ **2** and NH_4_ **5**) show isostructurality with their equivalent potassium structures. For anion **3**, the ammonium and Rb salt forms are also found to be isostructural, although unfortunately no other Rb salt structures are available for comparison. For the isostructural pair of **5**, all structural descriptors are very similar, but for the **2** and **3** pairs there are small differences in, for example, the rotation of the sulfonate groups that allow the ammonium cations to make less formal contacts than do the K or Rb ions. No sodium salt is found to be isostructural with an ammonium salt, but use of the ‘crystal packing similarity’ tool within *Mercury* did highlight that the azo anions of NH_4_ **3** do adopt similar packing to the chemically different azo anions of both Na**5** and Ag**5**, although this similarity only holds within a single hydro­phobic layer, with neighbouring layers behaving differently. The ammonium salt of Orange G shows similar single-layer-matching behaviour with its Na and mixed Na/K and Na/Rb salt forms. Overall there is thus a high propensity for ammonium salt forms of sulfonated azo dyes to be isostructural with the equivalent K or Rb forms, a propensity which is aided by small amounts of flexibility in the structures that allows for the different coordinating abilities of the cations. Little evidence is found for isostructural relationships between the salt forms of sulfon­ated azo dyes of ammonium salts and the equivalent sodium salts. Here, the smaller radius of the Na^+^ ion is perhaps hard to offset against the lower coordination number of sodium. However, the similarity of anion packing found, for instance, within single layers for NH_4_ **3** (and hence Rb**3**) with Na**5** and Ag**5** may indicate that isostructurality is possible and may be found if a larger sample of structures was available.

## Supplementary Material

Crystal structure: contains datablock(s) NH41, NH42, NH43, NH44, NH45, K1, K3, K4, K5, global. DOI: 10.1107/S2053229624001293/dg3051sup1.cif

Structure factors: contains datablock(s) NH41. DOI: 10.1107/S2053229624001293/dg3051NH41sup2.hkl

Structure factors: contains datablock(s) NH42. DOI: 10.1107/S2053229624001293/dg3051NH42sup3.hkl

Structure factors: contains datablock(s) NH43. DOI: 10.1107/S2053229624001293/dg3051NH43sup4.hkl

Structure factors: contains datablock(s) NH44. DOI: 10.1107/S2053229624001293/dg3051NH44sup5.hkl

Structure factors: contains datablock(s) NH45. DOI: 10.1107/S2053229624001293/dg3051NH45sup6.hkl

Structure factors: contains datablock(s) K1. DOI: 10.1107/S2053229624001293/dg3051K1sup7.hkl

Structure factors: contains datablock(s) K3. DOI: 10.1107/S2053229624001293/dg3051K3sup8.hkl

Structure factors: contains datablock(s) K4. DOI: 10.1107/S2053229624001293/dg3051K4sup9.hkl

Structure factors: contains datablock(s) K5. DOI: 10.1107/S2053229624001293/dg3051K5sup10.hkl

Supporting information file. DOI: 10.1107/S2053229624001293/dg3051NH41sup11.cml

Supporting information file. DOI: 10.1107/S2053229624001293/dg3051NH42sup12.cml

Supporting information file. DOI: 10.1107/S2053229624001293/dg3051NH43sup13.cml

Supporting information file. DOI: 10.1107/S2053229624001293/dg3051NH44sup14.cml

Supporting information file. DOI: 10.1107/S2053229624001293/dg3051NH45sup15.cml

CCDC references: 2331925, 2331924, 2331923, 2331922, 2331921, 2331920, 2331919, 2331918, 2331917

## Figures and Tables

**Figure 7:**
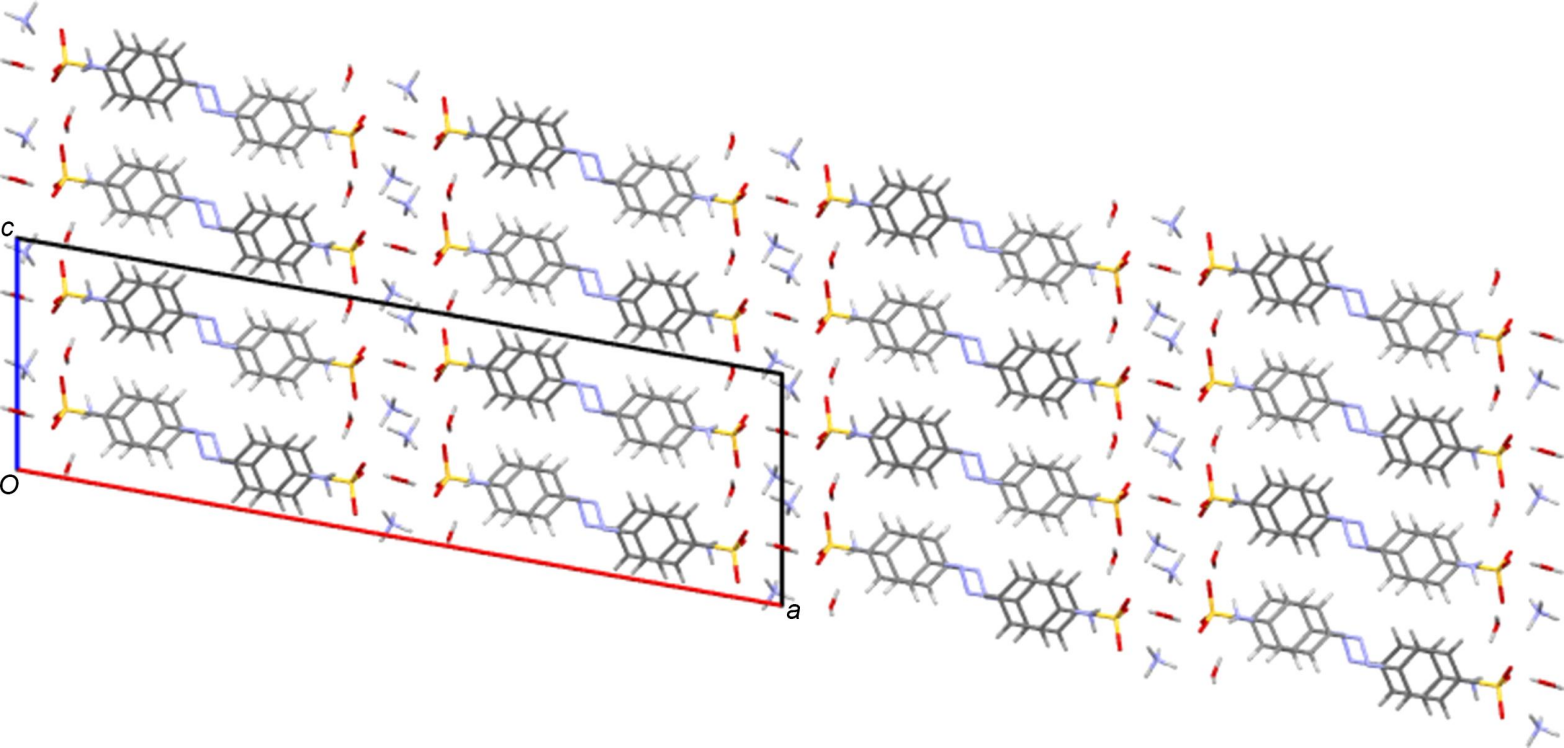
Packed structure of NH_4_ **3** viewed along the *b* axis. Despite the twisted nature of the azo anion, this structure has a similar simple layering structure with alternating hydro­phobic (aryl­azo) and hydro­philic (cation and water) layers as the planar species. Here the layers lie parallel to the *bc* plane.

**Table:** H atoms were treated by a mixture of independent and constrained refinement. The absorption correction was multi-scan for NH_4_ **1** (*CrysAlis PRO*; Rigaku OD, 2019 ▸) and K**1** (*SADABS*; Bruker, 2012 ▸).

	NH_4_ **1**	NH_4_ **2**	NH_4_ **3**
Crystal data			
Chemical formula	NH_4_^+^·C_12_H_9_N_2_O_4_S^−^	NH_4_^+^·C_12_H_9_N_2_O_5_S^−^·2H_2_O	NH_4_^+^·C_12_H_10_N_3_O_3_S^−^·1.5H_2_O
*M*_r_	295.31	347.34	321.35
Crystal system, space group	Monoclinic, *P*2_1_	Triclinic, *P* 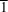	Monoclinic, *C*2/*c*
Temperature (K)	100	123	123
*a*, *b*, *c* (Å)	5.8163 (1), 6.9218 (1), 15.6295 (2)	8.2876 (1), 10.6404 (1), 17.4834 (3)	35.1636 (15), 7.8905 (3), 10.4972 (5)
α, β, γ (°)	90, 93.354 (1), 90	89.734 (1), 84.793 (1), 86.146 (1)	90, 100.091 (2), 90
*V* (Å^3^)	628.15 (2)	1531.91 (4)	2867.5 (2)
*Z*	2	4	8
Radiation type	Cu *K*α	Mo *K*α	Mo *K*α
μ (mm^−1^)	2.48	0.25	0.25
Crystal size (mm)	0.22 × 0.12 × 0.06	0.42 × 0.40 × 0.10	0.35 × 0.30 × 0.05

Data collection			
Diffractometer	Rigaku Synergy-i	Enraf–Nonius KappaCCD	Enraf–Nonius KappaCCD
*T*_min_, *T*_max_	0.859, 1.000	–	–
No. of measured, independent and observed [*I* > 2σ(*I*)] reflections	10144, 2345, 2336	15978, 8209, 6704	6133, 3271, 1857
*R*_int_	0.054	0.021	0.074
(sin θ/λ)_max_ (Å^−1^)	0.616	0.685	0.649

Refinement			
*R*[*F* ^2^ > 2σ(*F* ^2^)], *wR*(*F* ^2^), *S*	0.029, 0.084, 1.08	0.036, 0.094, 1.02	0.049, 0.105, 1.02
No. of reflections	2345	8209	3271
No. of parameters	203	495	231
No. of restraints	1	1	1
Δρ_max_, Δρ_min_ (e Å^−3^)	0.26, −0.33	0.58, −0.41	0.26, −0.46
Absolute structure	Refined as an inversion twin.	–	–
Absolute structure parameter	0.00 (2)	–	–

**Table:** 

	NH_4_ **4**	NH_4_ **5**	K**1**
Crystal data			
Chemical formula	NH_4_^+^·C_16_H_18_N_3_O_5_S^−^·H_2_O	NH_4_^+^·C_12_H_9_N_2_O_4_S^−^	[K(C_12_H_9_N_2_O_4_S)(H_2_O)]
*M*_r_	400.45	295.31	334.39
Crystal system, space group	Triclinic, *P* 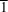	Orthorhombic, *P* *c* *c* *n*	Triclinic, *P* 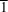
Temperature (K)	123	123	150
*a*, *b*, *c* (Å)	8.4933 (1), 13.0977 (2), 17.1657 (3)	12.6592 (3), 28.3597 (7), 7.1268 (2)	5.9620 (7), 7.2033 (11), 31.929 (5)
α, β, γ (°)	90.970 (1), 103.180 (1), 95.132 (1)	90, 90, 90	83.852 (14), 86.361 (15), 88.868 (15)
*V* (Å^3^)	1850.43 (5)	2558.60 (11)	1360.5 (3)
*Z*	4	8	4
Radiation type	Mo *K*α	Mo *K*α	Synchrotron, λ = 0.689 Å
μ (mm^−1^)	0.22	0.27	0.51
Crystal size (mm)	0.5 × 0.5 × 0.15	0.30 × 0.10 × 0.05	0.20 × 0.14 × 0.03

Data collection			
Diffractometer	Enraf–Nonius KappaCCD	Enraf–Nonius KappaCCD	Bruker APEXII CCD
*T*_min_, *T*_max_	–	–	0.751, 1.000
No. of measured, independent and observed [*I* > 2σ(*I*)] reflections	16736, 8841, 5911	5346, 2923, 1850	10143, 5691, 4405
*R*_int_	0.043	0.069	0.032
(sin θ/λ)_max_ (Å^−1^)	0.660	0.649	0.636

Refinement			
*R*[*F* ^2^ > 2σ(*F* ^2^)], *wR*(*F* ^2^), *S*	0.047, 0.118, 1.04	0.051, 0.118, 1.03	0.060, 0.180, 1.02
No. of reflections	8841	2923	5691
No. of parameters	550	201	403
No. of restraints	8	0	6
Δρ_max_, Δρ_min_ (e Å^−3^)	0.57, −0.42	0.29, −0.45	0.59, −0.67

**Table:** 

	K**3**	K**4**	K**5**
Crystal data			
Chemical formula	[K(C_12_H_10_N_3_O_3_S)(H_2_O)_2_]	[K(C_16_H_18_N_3_O_5_S)(H_2_O)_2_]	[K(C_12_H_9_N_2_O_4_S)]
*M*_r_	351.42	439.52	316.37
Crystal system, space group	Triclinic, *P* 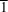	Monoclinic, *P*2_1_/*n*	Orthorhombic, *P* *c* *c* *n*
Temperature (K)	123	123	123
*a*, *b*, *c* (Å)	13.3058 (2), 13.6247 (2), 18.4664 (3)	9.4006 (2), 12.1583 (3), 34.4743 (9)	12.5535 (2), 27.9698 (5), 6.9982 (1)
α, β, γ (°)	88.373 (1), 73.971 (1), 66.313 (1)	90, 95.496 (1), 90	90, 90, 90
*V* (Å^3^)	2933.52 (8)	3922.14 (16)	2457.20 (7)
*Z*	8	8	8
Radiation type	Mo *K*α	Mo *K*α	Mo *K*α
μ (mm^−1^)	0.53	0.42	0.62
Crystal size (mm)	0.7 × 0.3 × 0.02	0.20 × 0.18 × 0.08	0.70 × 0.08 × 0.04

Data collection			
Diffractometer	Enraf–Nonius KappaCCD	Enraf–Nonius KappaCCD	Enraf–Nonius KappaCCD
No. of measured, independent and observed [*I* > 2σ(*I*)] reflections	26000, 13392, 10848	14501, 7971, 4387	5174, 2802, 1971
*R*_int_	0.023	0.084	0.056
(sin θ/λ)_max_ (Å^−1^)	0.649	0.628	0.648

Refinement			
*R*[*F* ^2^ > 2σ(*F* ^2^)], *wR*(*F* ^2^), *S*	0.036, 0.098, 1.03	0.060, 0.102, 1.01	0.042, 0.092, 1.05
No. of reflections	13392	7971	2802
No. of parameters	895	560	185
No. of restraints	24	112	1
Δρ_max_, Δρ_min_ (e Å^−3^)	0.80, −0.79	0.37, −0.34	0.39, −0.41

Computer programs: *CrysAlis PRO* (Rigaku OD, 2019 ▸), *DENZO* and *COLLECT* (Otwinowski & Minor, 1997 ▸), *APEX2*, *SAINT* and *SADABS* (Bruker, 2012 ▸), *SHELXT* (Sheldrick, 2015*a* ▸), *SIR92* (Altomare *et al.*, 1994 ▸) and *SHELXL2018* (Sheldrick, 2015*b* ▸) in *WinGX* (Farrugia, 2012 ▸).

**Table 2:** Hydrogen-bond geometry (Å, °) for NH_4_ **1** [Chem scheme1]

*D*—H⋯*A*	*D*—H	H⋯*A*	*D*⋯*A*	*D*—H⋯*A*
N3—H4N⋯O2^i^	0.91 (5)	2.30 (5)	2.940 (4)	127 (4)
N3—H4N⋯O1^ii^	0.91 (5)	2.21 (5)	2.969 (3)	141 (4)
N3—H3N⋯O3	0.86 (5)	1.96 (5)	2.808 (3)	170 (4)
N3—H1N⋯O1^iii^	0.86 (4)	2.03 (5)	2.889 (4)	176 (4)
N3—H2N⋯O4^iv^	0.88 (4)	2.10 (4)	2.946 (3)	159 (4)
O4—H1*H*⋯O2^v^	0.87 (5)	1.95 (5)	2.802 (3)	167 (4)

Symmetry codes: (i) 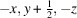 ; (ii) 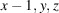 ; (iii) 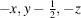 ; (iv) 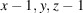 ; (v) 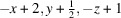 .

**Table 3:** Hydrogen-bond geometry (Å, °) for NH_4_ **2** [Chem scheme1]

*D*—H⋯*A*	*D*—H	H⋯*A*	*D*⋯*A*	*D*—H⋯*A*
O4—H1*H*⋯O4*W*	0.84 (2)	1.81 (2)	2.6357 (17)	170 (2)
O5—H2*H*⋯N1	0.98 (3)	1.65 (3)	2.5535 (16)	151 (2)
O9—H3*H*⋯O2*W*	0.85 (2)	1.83 (2)	2.6673 (16)	168 (2)
O10—H4*H*⋯N3	0.93 (3)	1.69 (3)	2.5420 (16)	149 (2)
N5—H1N⋯O1^i^	0.80 (2)	2.16 (2)	2.8858 (17)	151.1 (18)
N5—H1N⋯O1*W* ^ii^	0.80 (2)	2.627 (19)	3.034 (2)	113.3 (15)
N5—H2N⋯O1	0.92 (2)	1.94 (2)	2.8403 (18)	168 (2)
N5—H3N⋯O3*W* ^iii^	0.94 (2)	1.91 (2)	2.8488 (17)	172 (2)
N5—H4N⋯O6^iv^	0.89 (2)	2.01 (2)	2.8425 (16)	156 (2)
N6—H5N⋯S2^v^	0.90 (2)	2.99 (2)	3.7547 (15)	143.5 (17)
N6—H5N⋯O7^v^	0.90 (2)	1.99 (2)	2.8804 (18)	168 (2)
N6—H6N⋯O1*W*	0.92 (2)	1.90 (2)	2.8027 (18)	169.7 (19)
N6—H7N⋯O3*W* ^vi^	0.92 (2)	1.96 (2)	2.8733 (19)	170.4 (19)
N6—H8N⋯O2^vii^	0.84 (2)	2.42 (2)	2.9090 (18)	117.8 (18)
N6—H8N⋯O3^v^	0.84 (2)	2.36 (2)	2.9153 (17)	123.8 (19)
O1*W*—H2*W*⋯O4^viii^	0.84 (3)	2.01 (3)	2.8150 (16)	162 (2)
O1*W*—H1*W*⋯O8	0.82 (3)	1.98 (3)	2.7858 (17)	166 (2)
O2*W*—H3*W*⋯O3^viii^	0.83 (3)	2.04 (3)	2.8639 (16)	171 (2)
O2*W*—H4*W*⋯O8^vi^	0.87 (1)	1.94 (1)	2.7918 (17)	169 (2)
O3*W*—H5*W*⋯O7^viii^	0.88 (3)	1.83 (3)	2.7106 (16)	179 (2)
O3*W*—H6*W*⋯O9	0.95 (3)	2.04 (3)	2.8347 (15)	140 (2)
O4*W*—H7*W*⋯O2^ix^	0.81 (2)	1.94 (2)	2.7482 (17)	175 (2)
O4*W*—H8*W*⋯O6^ix^	0.81 (3)	2.01 (3)	2.8128 (16)	171 (2)

Symmetry codes: (i) 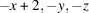 ; (ii) 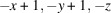 ; (iii) 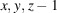 ; (iv) 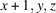 ; (v) 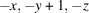 ; (vi) 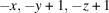 ; (vii) 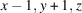 ; (viii) 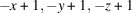 ; (ix) 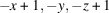 .

**Table 4:** Hydrogen-bond geometry (Å, °) for NH_4_ **3** [Chem scheme1]

*D*—H⋯*A*	*D*—H	H⋯*A*	*D*⋯*A*	*D*—H⋯*A*
N4—H1N⋯O2*W*	0.90 (3)	2.00 (3)	2.850 (3)	157 (2)
N4—H2N⋯O1^i^	0.94 (3)	1.93 (3)	2.862 (3)	167 (3)
N4—H2N⋯O1*W* ^i^	0.94 (3)	2.57 (3)	3.039 (3)	111 (2)
N4—H3N⋯O2^ii^	0.86 (3)	2.14 (3)	2.963 (3)	161 (3)
N4—H3N⋯O3^ii^	0.86 (3)	2.66 (3)	3.351 (3)	139 (3)
N4—H4N⋯O3^iii^	0.98 (4)	2.07 (4)	2.987 (3)	155 (3)
N3—H5N⋯O1*W* ^i^	0.85 (3)	2.23 (3)	3.067 (3)	168 (2)
N3—H6N⋯O2^ii^	0.86 (3)	2.17 (3)	3.015 (3)	165 (2)
O1*W*—H1*W*⋯N3^ii^	0.83 (3)	2.21 (3)	3.011 (3)	162 (3)
O1*W*—H2*W*⋯O3^iv^	0.88 (3)	1.99 (3)	2.861 (3)	169 (3)
O2*W*—H3*W*⋯O1^v^	0.87 (1)	1.97 (1)	2.792 (3)	159 (3)

Symmetry codes: (i) 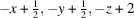 ; (ii) 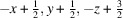 ; (iii) 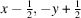 , 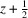 ; (iv) 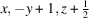 ; (v) 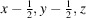 .

**Table 5:** Hydrogen-bond geometry (Å, °) for NH_4_ **4** [Chem scheme1]

*D*—H⋯*A*	*D*—H	H⋯*A*	*D*⋯*A*	*D*—H⋯*A*
O1*W*—H1*W*⋯O1^i^	0.88 (1)	1.93 (1)	2.802 (2)	176 (3)
O1*W*—H2*W*⋯O5^ii^	0.88 (1)	1.84 (1)	2.711 (2)	168 (2)
O2*W*—H3*W*⋯O1*A* ^ii^	0.91 (1)	2.05 (1)	2.945 (2)	172 (3)
O2*W*—H4*W*⋯O2*W* ^iii^	0.89 (1)	2.29 (4)	3.031 (5)	141 (6)
O4—H1*H*⋯O3*A* ^iv^	0.84 (3)	2.05 (3)	2.885 (2)	169 (3)
O5—H2*H*⋯O1*A*	0.81 (3)	1.90 (3)	2.698 (2)	169 (3)
O4*A*—H3*H*⋯O1^i^	0.80 (3)	1.96 (3)	2.756 (2)	177 (3)
O5*A*—H4*H*⋯O4*A*	0.97 (3)	1.79 (3)	2.734 (2)	165 (3)
N4—H1N⋯O3	0.87 (3)	2.09 (3)	2.957 (3)	176 (2)
N4—H2N⋯O2^v^	0.95 (3)	1.92 (3)	2.841 (3)	163 (2)
N4—H3N⋯O3*A* ^vi^	0.89 (3)	2.05 (3)	2.923 (3)	167 (2)
N4—H4N⋯O1*W*	1.13 (4)	1.69 (4)	2.818 (3)	176 (3)
N5—H5N⋯O2*A*	0.91 (3)	1.89 (3)	2.784 (3)	164 (2)
N5—H6N⋯O5*A* ^vii^	0.89 (4)	2.21 (4)	2.935 (3)	138 (3)
N5—H6N⋯O1*W* ^viii^	0.89 (4)	2.51 (4)	3.094 (3)	123 (3)
N5—H7N⋯O3^ix^	0.96 (4)	2.03 (4)	2.952 (3)	159 (3)
N5—H8N⋯O4^x^	0.96 (3)	2.00 (3)	2.943 (3)	168 (3)

Symmetry codes: (i) 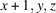 ; (ii) 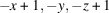 ; (iii) 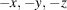 ; (iv) 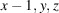 ; (v) 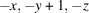 ; (vi) 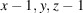 ; (vii) 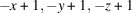 ; (viii) 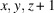 ; (ix) 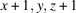 ; (x) 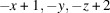 .

**Table 6:** Hydrogen-bond geometry (Å, °) for NH_4_ **5** [Chem scheme1]

*D*—H⋯*A*	*D*—H	H⋯*A*	*D*⋯*A*	*D*—H⋯*A*
N3—H1N⋯O2^i^	0.90 (4)	2.48 (3)	3.002 (4)	118 (3)
N3—H1N⋯O4^ii^	0.90 (4)	2.35 (3)	2.913 (4)	121 (3)
N3—H1N⋯O4^iii^	0.90 (4)	2.50 (4)	3.162 (4)	131 (3)
N3—H2N⋯O1	0.96 (4)	1.94 (4)	2.893 (3)	171 (3)
N3—H3N⋯O1^iv^	0.89 (3)	1.97 (3)	2.822 (3)	159 (3)
N3—H4N⋯O3^v^	0.90 (4)	1.97 (4)	2.831 (3)	160 (3)
O4—H1*H*⋯O2^vi^	0.82 (4)	2.00 (4)	2.707 (3)	144 (4)

Symmetry codes: (i) 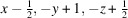 ; (ii) 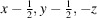 ; (iii) 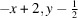 , 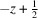 ; (iv) 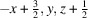 ; (v) 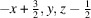 ; (vi) 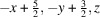 .

**Table 7:** Selected bond lengths (Å) for K**1** [Chem scheme1]

K1—O1	2.642 (2)	K2—O5	2.625 (2)
K1—O2*W* ^i^	2.688 (3)	K2—O2*W*	2.713 (2)
K1—O2^ii^	2.728 (2)	K2—O7^ii^	2.736 (2)
K1—O6	2.829 (3)	K2—O1^ii^	2.769 (2)
K1—O8^iii^	2.957 (2)	K2—O8^v^	2.904 (2)
K1—O7	3.027 (2)	K2—O3^ii^	3.020 (2)
K1—O4^iv^	3.116 (2)	K2—O1*W*	3.050 (3)
K1—O1*W* ^i^	3.149 (2)		

Symmetry codes: (i) 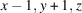 ; (ii) 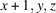 ; (iii) 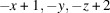 ; (iv) 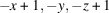 ; (v) 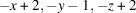 .

**Table 8:** Hydrogen-bond geometry (Å, °) for K**1** [Chem scheme1]

*D*—H⋯*A*	*D*—H	H⋯*A*	*D*⋯*A*	*D*—H⋯*A*
O4—H4*H*⋯O1*W* ^vi^	0.88 (1)	1.83 (2)	2.676 (3)	160 (3)
O8—H8*H*⋯O6^vii^	0.88 (1)	1.93 (3)	2.748 (3)	155 (5)
O1*W*—H1*W*⋯O3^ii^	0.87 (1)	1.95 (2)	2.801 (3)	165 (4)
O1*W*—H2*W*⋯O2^viii^	0.88 (1)	1.93 (1)	2.803 (4)	177 (5)
O2*W*—H3*W*⋯O3^ix^	0.88 (1)	2.20 (3)	2.866 (3)	133 (4)
O2*W*—H3*W*⋯O4^vi^	0.88 (1)	2.29 (3)	2.905 (3)	127 (3)
O2*W*—H4*W*⋯O5^ii^	0.88 (1)	1.92 (2)	2.778 (3)	165 (4)

Symmetry codes: (ii) 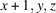 ; (vi) 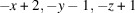 ; (vii) 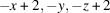 ; (viii) 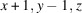 ; (ix) 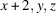 .

**Table 9:** Selected bond lengths (Å) for K**3** [Chem scheme1]

K1—O2	2.6531 (14)	K3—O3*C*	2.649 (2)
K1—O1*A* ^i^	2.6570 (14)	K3—O1*B*	2.7285 (16)
K1—O6*W* ^ii^	2.7388 (15)	K3—O5*W* ^iv^	2.8713 (16)
K1—O7*W* ^ii^	2.7669 (15)	K3—O4*W*	2.8932 (16)
K1—O1*W*	2.7806 (15)	K3—O5*W*	2.9166 (15)
K1—O1*A*	2.8284 (14)	K3—O3*B*	3.2009 (19)
K1—O2*A*	2.9928 (15)	K3—O4*W* ^iv^	3.2971 (17)
K2—O3	2.7462 (14)	K4—O2*B* ^v^	2.6854 (15)
K2—O1*C*	2.7536 (17)	K4—O6*W*	2.7164 (15)
K2—O2*A*	2.7929 (14)	K4—O2*B*	2.7701 (15)
K2—O2*W*	2.8559 (16)	K4—O8*W*	2.8338 (16)
K2—O3*W* ^iii^	2.8666 (15)	K4—O7*W*	2.8395 (16)
K2—O3*W*	2.8901 (16)	K4—O3*C*	2.954 (2)
K2—O2	3.0708 (15)	K4—O2*C*	3.097 (2)
K2—O3*A*	3.1446 (16)	K4—O1*B*	3.1503 (16)
K3—O3	2.6206 (14)		

Symmetry codes: (i) 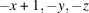 ; (ii) 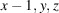 ; (iii) 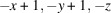 ; (iv) 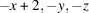 ; (v) 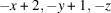 .

**Table 10:** Hydrogen-bond geometry (Å, °) for K**3** [Chem scheme1]

*D*—H⋯*A*	*D*—H	H⋯*A*	*D*⋯*A*	*D*—H⋯*A*
N3—H1N⋯O3*B* ^vi^	0.92 (3)	2.35 (3)	3.210 (2)	155 (2)
N3—H2N⋯O2*W* ^vi^	0.81 (3)	2.40 (3)	3.147 (2)	154 (2)
N3*A*—H3N⋯O8*W* ^vii^	0.82 (3)	2.27 (3)	3.016 (2)	153 (3)
N3*A*—H4N⋯O1^viii^	0.88 (3)	2.27 (3)	3.115 (2)	161 (2)
N3*B*—H5N⋯O2*C* ^ix^	0.90 (3)	2.50 (3)	3.384 (3)	167 (2)
N3*B*—H5N⋯O5*C* ^ix^	0.90 (3)	2.26 (3)	3.078 (14)	151 (2)
N3*B*—H6N⋯O1*W* ^vi^	0.88 (3)	2.21 (3)	3.065 (2)	164 (2)
N3*C*—H7N⋯O4*W* ^viii^	0.86 (3)	2.38 (3)	3.149 (2)	149 (2)
N3*C*—H8N⋯O3*A* ^vii^	0.87 (3)	2.53 (3)	3.330 (2)	154 (2)
O1*W*—H1*W*⋯N3*C* ^x^	0.87 (1)	2.62 (2)	3.428 (2)	156 (2)
O1*W*—H2*W*⋯O1*B* ^ii^	0.87 (1)	1.94 (1)	2.808 (2)	174 (2)
O2*W*—H3*W*⋯O1*C* ^iii^	0.87 (1)	2.14 (1)	2.963 (3)	160 (3)
O2*W*—H3*W*⋯O5*C* ^iii^	0.87 (1)	2.24 (3)	2.973 (18)	143 (2)
O2*W*—H4*W*⋯N3*B* ^vi^	0.87 (1)	2.56 (2)	3.322 (2)	148 (2)
O3*W*—H5*W*⋯N3^vi^	0.87 (1)	2.25 (2)	3.036 (2)	151 (3)
O3*W*—H6*W*⋯O3*B*	0.87 (1)	2.15 (2)	2.906 (2)	145 (2)
O4*W*—H7*W*⋯O1	0.87 (1)	2.03 (1)	2.879 (2)	166 (3)
O4*W*—H8*W*⋯O3*C* ^iv^	0.87 (1)	2.02 (1)	2.837 (2)	156 (2)
O4*W*—H8*W*⋯O6*C* ^iv^	0.87 (1)	2.27 (2)	3.126 (16)	170 (3)
O5*W*—H9*W*⋯O3^iv^	0.87 (1)	2.66 (2)	3.376 (2)	141 (2)
O5*W*—H10*W*⋯O3*A* ^iv^	0.89 (1)	1.93 (1)	2.807 (2)	169 (2)
O6*W*—H11*W*⋯O5*W*	0.87 (1)	2.11 (1)	2.924 (2)	158 (2)
O6*W*—H12*W*⋯O1^iv^	0.86 (1)	1.95 (1)	2.7885 (19)	164 (3)
O7*W*—H13*W*⋯O2*C* ^v^	0.87 (1)	2.02 (1)	2.896 (2)	177 (3)
O7*W*—H13*W*⋯O5*C* ^v^	0.87 (1)	1.88 (2)	2.698 (12)	156 (3)
O7*W*—H14*W*⋯O3*W* ^v^	0.88 (1)	2.22 (1)	3.085 (2)	169 (2)
O8*W*—H15*W*⋯O2*A* ^xi^	0.88 (1)	1.94 (1)	2.808 (2)	168 (2)

Symmetry codes: (ii) 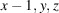 ; (iii) 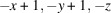 ; (iv) 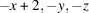 ; (v) 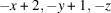 ; (vi) 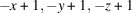 ; (vii) 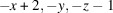 ; (viii) 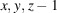 ; (ix) 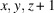 ; (x) 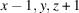 ; (xi) 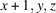 .

**Table 11:** Selected bond lengths (Å) for K**4** [Chem scheme1]

K1—O2*W* ^i^	2.738 (3)	K2—O5^v^	2.794 (3)
K1—O1*A* ^ii^	2.771 (3)	K2—O1*A*	2.800 (2)
K1—O1*W*	2.772 (3)	K2—O2*A* ^ii^	2.821 (2)
K1—O2*A*	2.786 (2)	K2—O1	2.862 (2)
K1—O3*W* ^iii^	2.798 (3)	K2—O1*W* ^ii^	2.898 (3)
K1—O4*A* ^iv^	2.820 (3)	K2—O3*A*	2.947 (2)
K1—O3*A*	2.919 (2)	K2—O2	3.073 (3)
K2—O2*W* ^i^	2.753 (3)		

Symmetry codes: (i) 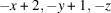 ; (ii) 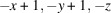 ; (iii) 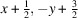 , 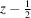 ; (iv) 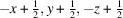 ; (v) 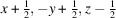 .

**Table 12:** Hydrogen-bond geometry (Å, °) for K**4** [Chem scheme1]

*D*—H⋯*A*	*D*—H	H⋯*A*	*D*⋯*A*	*D*—H⋯*A*
O4—H1*H*⋯O4*W*	0.87 (1)	1.84 (1)	2.703 (4)	171 (4)
O5—H2*H*⋯O4	0.88 (1)	1.85 (1)	2.719 (3)	173 (4)
O4*A*—H3*H*⋯O5*A*	0.87 (1)	1.86 (2)	2.695 (3)	161 (3)
O5*A*—H4*H*⋯O3*W*	0.88 (1)	1.93 (2)	2.738 (3)	154 (3)
O1*W*—H1*W*⋯O5*A* ^iii^	0.87 (1)	2.22 (2)	3.017 (3)	151 (3)
O1*W*—H2*W*⋯O4^iv^	0.87 (1)	2.08 (1)	2.906 (3)	158 (3)
O2*W*—H3*W*⋯O5^v^	0.87 (1)	1.93 (1)	2.791 (4)	170 (4)
O2*W*—H4*W*⋯O2	0.87 (1)	1.99 (2)	2.795 (3)	153 (3)
O3*W*—H5*W*⋯O3^vi^	0.87 (1)	1.93 (1)	2.786 (3)	167 (3)
O3*W*—H6*W*⋯O4*W* ^vii^	0.88 (1)	1.99 (1)	2.821 (4)	158 (3)
O4*W*—H7*W*⋯O4*A* ^viii^	0.88 (1)	1.94 (1)	2.789 (3)	163 (3)
O4*W*—H8*W*⋯O1^vi^	0.88 (1)	1.86 (1)	2.738 (3)	180 (4)

Symmetry codes: (iii) 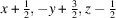 ; (iv) 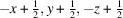 ; (v) 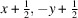 , 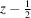 ; (vi) 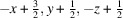 ; (vii) 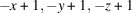 ; (viii) 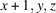 .

**Table 13:** Selected bond lengths (Å) for K**5** [Chem scheme1]

K1—O2^i^	2.7117 (18)	K1—O1	2.863 (2)
K1—O3^ii^	2.7511 (18)	K1—O2	2.970 (2)
K1—O4^iii^	2.7954 (19)	K1—O4^v^	3.0023 (19)
K1—O3^iv^	2.815 (2)		

Symmetry codes: (i) 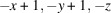 ; (ii) 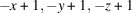 ; (iii) 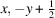 , 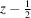 ; (iv) 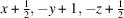 ; (v) 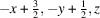 .

**Table 14:** Hydrogen-bond geometry (Å, °) for K**5** [Chem scheme1]

*D*—H⋯*A*	*D*—H	H⋯*A*	*D*⋯*A*	*D*—H⋯*A*
O4—H1*H*⋯O1^v^	0.87 (1)	2.02 (3)	2.732 (3)	139 (3)

Symmetry code: (v) 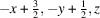 .

**Table:** 

	NH_4_ **1**	Na**1**	K**1**	NH_4_ **2**	Na**2**	K**2**	NH_4_ **3**	Na**3**	K**3**
CN	6	6	8, 7	6, 6	6	7, 7	6	6	7,8,8,8
CPD		1	3		2	1		2	2
H_2_O	0	2	1	2	2.5	2	1.5	2	2
Cation-to-SO_3_ inter­action	yes	yes	yes	yes	yes	yes	yes	yes	yes
Cation-to-tail inter­action	yes	no	yes	no	no	no	no	yes	no
Cation-to-water inter­action	no	yes	yes	yes	yes	yes	yes	yes	yes
Space group	*P*2_1_	*Pbcn*	*P* 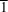	*P* 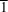	*C*2/*c*	*P* 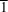	*C*2/*c*	*P*2_1_	*P* 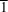
*a* (Å)	5.8163 (1)	14.383	5.9620 (7)	8.2876 (1)	35.036	8.177	35.1636 (15)	7.829	13.3058 (2)
*b* (Å)	6.9218 (1)	5.813	7.2033 (11)	10.6404 (1)	5.410	10.529	7.8905 (3)	5.784	13.6247 (2)
*c* (Å)	15.6295 (2)	32.891	31.929 (5)	17.4834 (3)	15.978	17.483	10.4972 (5)	16.486	18.4664 (3)
α (°)	90	90	83.852 (14)	89.734 (1)	90	89.82	90	90	88.373 (1)
β (°)	93.354 (1)	90	86.361 (15)	84.793 (1)	98.05	85.49	100.091 (2)	98.62	73.971 (1)
γ (°)	90	90	88.868 (15)	86.146 (1)	90	86.81	90	90	66.313 (1)
*V*/*Z* (Å^3^)	314.07	343.75	340.12	382.98	374.85	374.57	358.43	369.05	366.69

**Table:** 

	NH_4_ **4**	Na**4**	K**4**	NH_4_ **5**	Na**5**	K**5**
CN	5, 6	7,7	7, 8	7	6	7
CPD		2	2		2	3
H_2_O	1	1.5	2	0	2	0
Cation-to-SO_3_ inter­action	yes	yes	yes	yes	yes	yes
Cation-to-tail inter­action	yes	yes	yes	yes	yes	yes
Cation-to-water inter­action	yes	yes	yes	no	yes	no
Space group	*P* 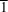	*P* 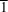	*P*2_1_/*n*	*Pccn*	*Pbca*	*Pccn*
*a* (Å)	8.4930 (1)	9.691	9.4006 (2)	12.6590 (2)	7.123	12.5535 (2)
*b* (Å)	13.0980 (2)	11.487	12.1583 (3)	28.3600 (3)	11.940	27.9698 (5)
*c* (Å)	17.1660 (3)	16.605	34.4743 (9)	7.1270 (7)	32.561	6.9982 (1)
α (°)	90.970 (1)	93.73	90	90	90	90
β (°)	103.180 (1)	92.76	95.496 (1)	90	90	90
γ (°)	95.132 (1)	95.37	90	90	90	90
*V*/*Z* (Å^3^)	462.61	458.04	490.27	319.82	346.14	307.15

Notes: CN = coordination number; CPD = coordination polymer dimensionality; *V*/*Z* = unit-cell volume divided by *Z*.

**Table 16:** Unit-cell dimensions of selected salt forms of Orange G (OG) Data taken from Ojala *et al.* (1994 ▸) and from Kennedy *et al.* (2006 ▸).

	NH_4_OG	AgOG	NaOG	Na/KOG	Na/RbOG
Space group	*P* 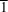	*P* 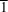	*P* 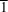	*P* 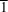	*P* 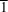
*a* (Å)	9.165	8.870	8.900	8.991	9.025
*b* (Å)	10.149	10.678	10.470	10.401	10.654
*c* (Å)	12.623	13.273	13.735	14.996	15.077
α (°)	87.43	73.56	73.49	83.35	82.30
β (°)	88.07	77.19	79.29	85.05	84.92
γ (°)	71.00	71.66	69.50	70.23	69.39
